# Iridium-catalyzed asymmetric ring-opening reactions of oxabicyclic alkenes with secondary amine nucleophiles

**DOI:** 10.3762/bjoc.5.53

**Published:** 2009-10-09

**Authors:** Dingqiao Yang, Ping Hu, Yuhua Long, Yujuan Wu, Heping Zeng, Hui Wang, Xiongjun Zuo

**Affiliations:** 1 School of Chemistry and Environment, South China Normal University, Guangzhou 510006, People’s Republic of China, Fax: (+86)-20-39310187; Phone: (+86)-20-85210087

**Keywords:** chiral bisphosphine ligand, iridium catalyst, oxabicyclic alkenes, ring-opening reaction

## Abstract

Iridium-catalyzed asymmetric ring-opening reactions of oxabicyclic alkenes with various aliphatic and aromatic secondary amines are reported for the first time. The reaction gave the corresponding *trans*-1,2-dihydronaphthalenol derivatives in good yields with moderate enantioselectivities in the presence of 2.5 mol % [Ir(COD)Cl]_2_ and 5 mol % bisphosphine ligand (*S*)-*p*-Tol-BINAP. The *trans*-configuration of **3f** was confirmed by X-ray crystallography.

## Introduction

Substituted dihydronaphthalenes are important molecules with different biological activities [1–5]. Therefore, the synthesis of these molecules has been an attractive research topic in recent years. Among the many reported methods for the preparation of the dihydronaphthalene skeleton, transition metal-catalyzed asymmetric ring-opening (ARO) of oxabicyclic alkenes is one of the most attractive because this reaction could potentially create two chiral centers in a single step.

Pioneering work in this field was first described by Caple et al. [6] and the group of Lautens [7–9]. In the past decades, the group of Lautens and others reported rhodium-catalyzed asymmetric ring-opening of oxabenzonorbornadiene with a wide range of nucleophiles including thiols [10], phenols [11], organoboronic acids [12–13], dialkylzincs [14–15], carboxylates [16], sulfur nucleophiles [17], and various amines [18–19].

In addition to rhodium catalysts, other transition metal catalysts may be used for asymmetric ring-opening reactions of oxabicyclic alkenes. These include complexes of copper [20–25], palladium [14–15,26–31], iron [32], and nickel [33–36]. Recently, we reported for the first time iridium-catalyzed asymmetric ring-opening of *N*-Boc-azabenzonorbornadiene with a wide range of secondary amines [37].

In this article, we will report ARO reactions of oxabicyclic alkenes with aliphatic and aromatic secondary amine nucleophiles using iridium-complex catalysts, which provide a fast and efficient access to chiral molecules with the dihydronaphthalene skeleton.

## Results and Discussion

**Scheme 1:**
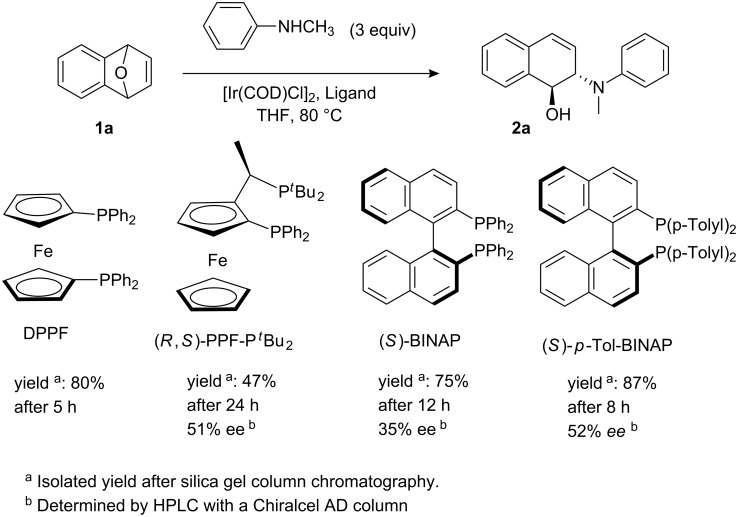
Identification of optimal chiral ligand for iridium-catalyzed asymmetric ring-opening of oxabenzonorbornadiene **1a** with *N*-methylaniline.

The ARO reaction involves many components of the chemical agents; we first attempted to optimize the ligand to iridium catalyst system (Scheme 1). In our initial experiments, we chose an achiral 1,1′-bis(diphenylphosphino)ferrocene (DPPF) ligand to validate the catalytic activity of the iridium complex. The product **2a** was obtained in high yield (80%) in the presence of 2.5 mol % [Ir(COD)Cl]_2_ and 5 mol % DPPF in THF after 5 h.

Encouraged by this result, we then ran the asymmetric version of the same reaction using different kinds of chiral bisphosphine ligands as shown in Scheme 1. We first examined ferrocenyl bisphosphine ligand, (*R*,*S*)-PPF-P*^t^*Bu_2_, which was identified as the ligand giving the best reactivity and excellent enantioselectivity in Rh-catalyzed system [11–15]. Unfortunately, the desired ring-opened product **2a** was obtained only in low yield (47%) with reasonable enantioselectivity (51% ee) in the iridium-catalyzed system. This suggested that (*R*,*S*)-PPF-P*^t^*Bu_2_ was not an ideal ligand in iridium-catalyzed reactions, which prompted us to screen other ligands. Among the several chiral ligands we had tested, (*S*)-BINAP and (*S*)-*p*-Tol-BINAP were found to give better yields and reasonable enantioselectivities. Moreover, in the case of (*S*)-*p*-Tol-BINAP, the enantioselectivity is slightly higher than for (*S*)-BINAP (52% ee vs 35% ee); therefore, we decided to use (*S*)-*p*-Tol-BINAP as the ligand for this ring-opening reaction.

Using (*S*)-*p*-Tol-BINAP as our standard ligand, we next investigated the effect of different solvents on reactivity and enantioselectivity (Table 1). Among the solvents examined for asymmetric ring-opening of oxabenzonorbornadiene **1a**, THF was found to be the best in terms of yield and enantioselectivity (Table 1, entry 6). Reactions in toluene and dioxane afforded the desired product **2a** in 70% and 76% yields, respectively. However, the enantioselectivities were slightly lower (Table 1, entries 3 and 4). On the other hand, temperature had a remarkable impact on reactivity and enantioselectivity. At room temperature, the reaction failed to give the corresponding ring-opened product **2a** (Table 1, entry 7). A sluggish reaction was observed at 65 °C (Table 1, entry 8), whereas at a higher temperature, such as 100 °C, the reaction gave a better yield and moderate enantioselectivity (Table 1, entry 9).

**Table 1:** Screening conditions for iridium-catalyzed asymmetric ring-opening of oxabenzonorbornadiene **1a** with *N*-methylaniline.^a^

Entry	Solvent	Temperature (°C)	Time (h)	Yield (%)^b^	ee (%)^c^

1	DME	100	12	48	16
2	CH_3_CN	90	12	36	21
3	Toluene	110	8	70	34
4	Dioxane	110	8	76	46
5	THF	100	12	65	50
6	THF	80	8	87	52
7	THF	25	48	n.r.	–
8	THF	65	12	58	53
9	THF	100	5	89	46

^a^The reaction was carried out with **1a** (0.34 mmol) and 3.0 equiv of *N*-methylaniline (1.0 mmol) in a solvent (2.0 mL) in the presence of [Ir(COD)Cl]_2_ (2.5 mol %) and (*S*)-*p*-Tol-BINAP (5.0 mol %). ^b^Isolated yields after silica gel column chromatography. ^c^Determined by HPLC with a Chiralcel AD column.

Based on the above findings, we decided to use the following reaction condition as a standard to run the ARO reactions, which consisted of 2.5 mol % [Ir(COD)Cl]_2_, 5 mol % (*S*)-*p*-Tol-BINAP, and 3.0 equiv of substituted *N*-alkylaniline in THF at 80 °C. The results of the ARO of oxabenzonorbornadiene **1a** are shown in Table 2.

**Table 2:** Scope of ring-opening of oxabenzonorbornadiene **1a** with substituted *N*-alkylaniline.^a^

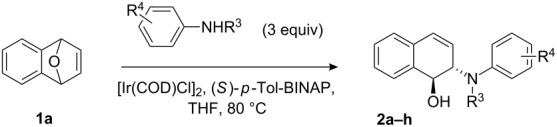						
Entry	R^3^	R^4^	Product	Time (h)	Yield (%)^b^	ee (%)^c^

1	CH_3_	H	**2a**	8	87	52
2	CH_2_CH_3_	H	**2b**	12	75	56
3	CH_2_CH=CH_2_	H	**2c**	24	48	74
4	Cyclohexyl	H	–	60	n.r.	–
5	CH_3_	2-Cl	–	24	n.r.	–
6	CH_3_	3-Cl	**2d**	24	56	72
7	CH_3_	4-Cl	**2e**	12	70	55
8	CH_3_	4-F	**2f**	12	65	51
9	CH_3_	4-Br	**2g**	12	71	65
10	CH_3_	4-NO_2_	–	60	n.r.	–
11	CH_3_	4-OCH_3_	**2h**	6	85	50

^a^The reaction was carried out with **1a** (0.34 mmol) and 3.0 equiv of substituted *N*-alkylaniline (1.0 mmol) in THF (2.0 mL) at 80 °C in the presence of [Ir(COD)Cl]_2_ (2.5 mol %) and (*S*)-*p*-Tol-BINAP (5.0 mol %). ^b^Isolated yields after silica gel column chromatography. ^c^Determined by HPLC with a Chiralcel AD column.

The results demonstrated that the size of the alkyl group on nitrogen in the nucleophile significantly influenced the reactivity and enantioselectivity. For instance, in the reaction of **1a** with *N*-methylaniline, which has a small group on *N*-atom, the ring-opening product **2a** was obtained in 87% yield and 52% ee (Table 2, entry 1), whereas in the reaction of **1a** with *N*-ethylaniline, which has a larger group on *N*-atom, the yield decreased to 75%, but the enantioselectivity was slightly increased (Table 2, entry 2). When *N*-allylaniline was used as a nucleophile, we obtained the product **2c** in 48% yield with 74% ee (Table 2, entry 3). The reaction using *N*-cyclohexylaniline failed to give any ring-opened product because of the steric hindrance of the bulky group (Table 2, entry 4).

We then investigated the effect of substituent groups on the aromatic ring of *N*-methylaniline. In 4-chloro-*N*-methylaniline, the reaction gave the desired product **2e** in high yield with moderate enantioselectivity (Table 2, entry 7). When 3-chloro-*N*-methylaniline was used, the corresponding product **2d** was obtained in slightly lower yield with slightly higher enantioselectivity (Table 2, entry 6). However, the reaction failed to give the expected product when *ortho*-substituted *N*-methylanilines were used (Table 2, entry 5). Various *para*-substituted *N*-methylanilines were shown to give the expected products in high yields with moderate enantioselectivities (Table 2, entries 8, 9 and 11). From Table 2, it was also found that the electronic property of the *N*-methylaniline had a significant impact on the reactivity. Electron-rich amines had higher reactivity than electron-deficient amines (Table 2, entries 1–3, 5, and 10).

To evaluate the scope of the reaction, we also examined various aliphatic secondary amines under optimized reaction conditions, and the results are summarized in Table 3. In most cases, aliphatic secondary amines reacted smoothly with **1a** to give the corresponding ring-opened products (**2i**–**l**) in high yields (up to 90%) with enantioselectivity ranging between 49% ee and 65% ee (Table 3, entries 5–8). We also found that the halide ions might play an important role in transition metal-catalyzed ARO reactions, in which the reactivity and enantioselectivity could be significantly improved by choosing a suitable halide ion. It was found that the reaction yields and enantioselectivities increased in the order of F<Cl<Br<I (Table 3, entries 2–5). But under the optimized reaction conditions, only a low yield (16%) was obtained without halide ion additives (Table 3, entry 1). Therefore, simply changing the halide ligand on the iridium catalyst from chloride to iodide or bromide leads to improvements in the reactivity and enantioselectivity. On the other hand, the difference in enantioselectivity between the fluoro and the iodo complexes is particularly striking with piperidine as the nucleophile. With the Ir–F catalyst, **2i** is formed in only 32% yield and 40% ee. Changing to the Ir–I complex gives **2i** in 90% yield and 49% ee (Table 3, entries 2 and 5).

**Table 3:** ARO of oxabenzonorbornadiene **1a** with various aliphatic secondary amines.^a^

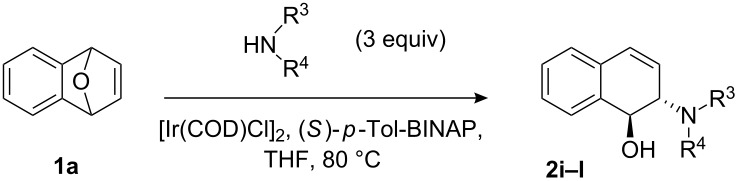						
Entry	HNR^3^R^4^	Additive^b^	Product	Time (h)	Yield (%)^c^	ee (%)^d^

1	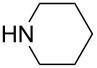	–	**2i**	24	16	45
2		NH_4_F		24	32	40
3		NH_4_Cl		24	45	43
4		NH_4_Br		24	60	45
5		NH_4_I		12	90	49
6	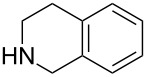	NH_4_I	**2j**	20	72	65
7	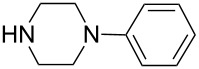	NH_4_I	**2k**	8	88	53
8	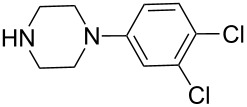	NH_4_I	**2l**	15	78	56

^a^The reaction was carried out with **1a** (0.34 mmol) and 3.0 equiv of aliphatic secondary amine (1.0 mmol) in THF (2.0 mL) at 80 °C in the presence of [Ir(COD)Cl]_2_ (2.5 mol %) and (*S*)-*p*-Tol-BINAP (5.0 mol %). ^b^Additive is 1.0 equiv relative to **1a**. ^c^Isolated yields after silica gel column chromatography. ^d^Determined by HPLC with a Chiralcel AD column.

Having examined a wide range of secondary amines, it was found that the sterically hindered nucleophiles enhanced the enantioselectivities (up to 89% ee). Inspired by this observation, we then examined more sterically hindered substrate **1b** or **1c** in the presence of 2.5 mol % [Ir(COD)Cl]_2_, 5 mol % (*S*)-*p*-Tol-BINAP, and 3.0 equiv of secondary amine nucleophiles in THF, at reflux. The results are summarized in Table 4 and Table 5.

The reactions of substrate **1b**, which contained methoxy groups on the 5- and 8-positions, with various secondary amines provided ring-opened products **3a**–**j** in yields from 65% to 83% and enantioselectivities from 41% to 89%. We further found that the substituent groups on the substrate did not apparently influence the yields, whereas the ee values increased, in some cases, to as high as 89% ee (Table 4, entry 6).

**Table 4:** ARO of oxabenzonorbornadiene **1b** with various secondary amines.^a^

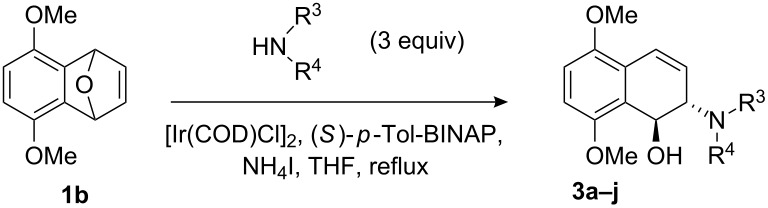					
Entry	HNR^3^R^4^	Product	Time (h)	Yield (%)^b^	ee (%)^c^

1	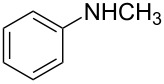	**3a**	12	81	76
2	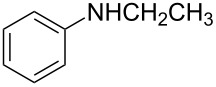	**3b**	20	70	81
3	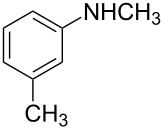	**3c**	8	68	43
4	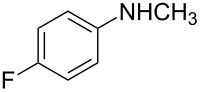	**3d**	12	65	45
5	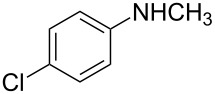	**3e**	12	74	54
6	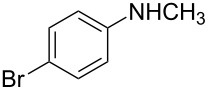	**3f**	12	70	89
7	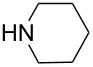	**3g**	6	83	41
8	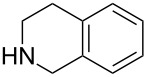	**3h**	12	71	63
9	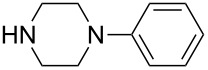	**3i**	12	79	54
10	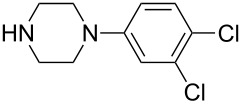	**3j**	12	75	56

^a^The reaction was carried out with **1b** (0.34 mmol) and 3.0 equiv of secondary amine (1.0 mmol) in THF (2.0 mL) at reflux in the presence of [Ir(COD)Cl]_2_ (2.5 mol %) and (*S*)-*p*-Tol-BINAP (5.0 mol %). The NH_4_I additive is 1.0 equiv relative to **1b**. ^b^Isolated yields after silica gel column chromatography. ^c^Determined by HPLC with a Chiralcel AD column.

**Table 5:** Iridium-catalyzed asymmetric ring-opening reaction of substrate **1c**.^a^

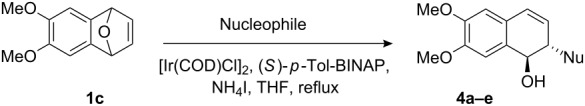						
Entry	Nucleophile	Ligand	Product	Time (h)	Yield (%)^b^	ee (%)^c^

1	2-Piperazin-1-ylbenzonitrile	(*S*)-*p*-Tol-BINAP	**4a**	24	70	37
2	4-Fluorophenylpiperazine	(*S*)-*p*-Tol-BINAP	**4b**	24	68	49
3	2-Fluorophenylpiperazine	(*S*)-*p*-Tol-BINAP	**4c**	24	73	38
4	1-(4-Methoxyphenyl)piperazine	(*S*)-*p*-Tol-BINAP	**4d**	24	62	59
5	3,4-Dichlorophenylpiperazine	(*S*)-*p*-Tol-BINAP	**4e**	24	27	16

^a^Conditions: [Ir(COD)Cl]_2_ (2.5 mol %) and ligand (5.0 mol %) were dissolved in THF and stirred for 10–20 min. Then NH_4_I was added and the mixture stirred for another 10–20 min. The substrate **1c** was added and the mixture heated to reflux. The nucleophiles were added on the first sign of reflux. ^b^Isolated yields after silica gel column chromatography. ^c^Determined by HPLC with a Chiralcel AD column or AD column.

**Figure 1:**
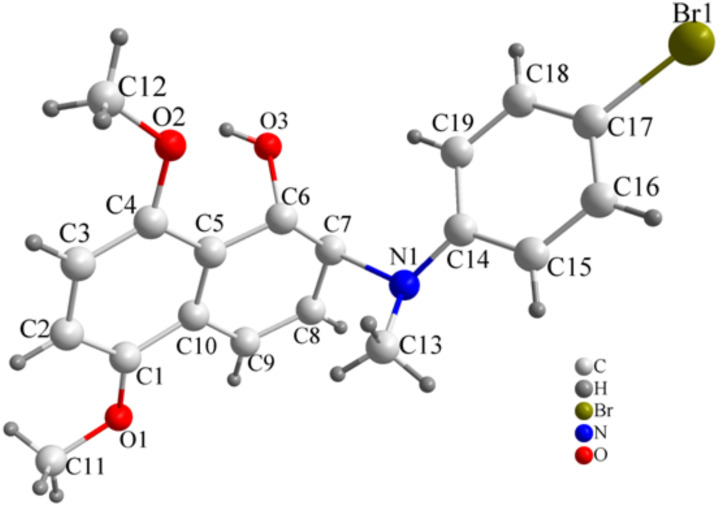
ORTEP plot for **3f**.

The absolute configuration of ring-opened product **3f** was demonstrated by X-ray crystallography. The single crystal was obtained by evaporation of the solvent from its solution in dichloromethane and petroleum ether. Its configuration was assigned as (1*S*,2*S*), and the hydroxyl group and 4-bromo-*N*-methylaniline group are in a *trans* relationship (Figure 1).

**Scheme 2:**
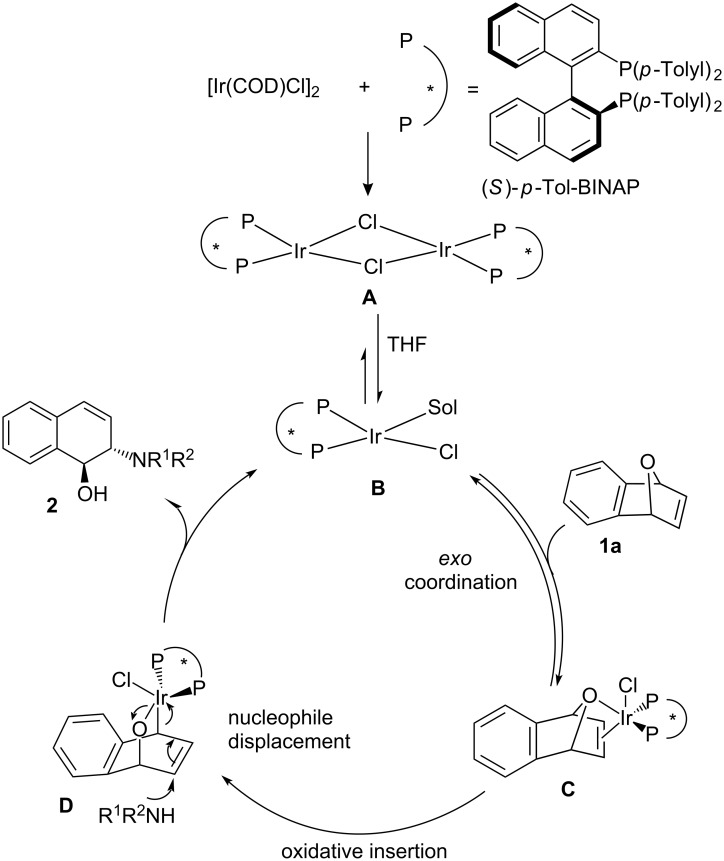
Proposed mechanism for the ARO of oxabenzonorbornadiene **1a** with secondary amine nucleophiles.

Based on our studies in this article, the reaction mechanism is proposed as shown in Scheme 2. The chiral dimeric iridium complex **A** is first formed. The dimer **A** is cleaved by solvation (THF) to become monomer **B**. The oxygen atom and the double bond of oxabenzonorbornadiene **1a** are then reversibly coordinated to the iridium center of the catalyst to give the intermediate **C**. Oxidative insertion of **C** in to the C–O bond forms **D**. Then, attack of the secondary amine nucleophile along with configurational inversion is proposed to occur in an S_N_2′ displacement of the iridium catalyst. The *trans*-1,2-dihydronaphthalenol product **2** is subsequently released and the iridium monomer **B** is regenerated.

## Conclusion

In summary, we have explored the iridium-catalyzed ARO reaction of oxabicyclic alkenes with *N*-alkylated anilines or *N*-substituted piperazine nucleophiles; the reactions gave the desired products in moderate to good yields with good enantioselectivities. The iridium-catalyzed ARO reactions described in this article featured lower cost compared with rhodium-catalyzed ARO reactions, which provided potential applications in asymmetric synthesis of chiral building blocks. The 1,2-*trans*-configuration of the product was confirmed by X-ray crystallography. The search for an optimized ligand aiming to enhance enantioselectivity and the extension of this reaction to other types of substrates and nucleophiles are currently under investigation in our labs.

## Experimental

**General procedure (I) for the asymmetric ring-opening reactions of oxabenzonorbornadiene 1a with substituted *N*-alkylaniline:** A 5.0 mL round-bottom flask fitted with a reflux condenser was flame-dried under a stream of nitrogen and cooled to room temperature. [Ir(COD)Cl]_2_ (5.8 mg, 2.5 mol %) and (*S*)-*p*-Tol-BINAP (10.7 mg, 5 mol %) were simultaneously added, followed by an addition of anhydrous tetrahydrofuran (2.0 mL). After they were stirred for about 10 min, oxabenzonorbornadiene **1a** (50.0 mg, 0.347 mmol) was added and the resulting mixture was heated to reflux. On the first sign of reflux, nucleophile (3.0 equiv to **1a**) was added. The temperature was continuously increased to 80 °C until the reaction was complete as determined by thin layer chromatography. The reaction mixture was then concentrated in vacuo and purified by column chromatography (silica gel: 200–300 mesh) to give the target product.

**(1*S*,2*S*)-2-[Methyl(phenyl)amino]-1,2-dihydronaphthalen-1-ol (2a):** Following the general procedure (**I**), **2a** was obtained as a colorless oil (76.0 mg, 87%). *R_f_* = 0.17 on silica gel (ethyl acetate:petroleum ether = 1:20, v/v). The ee was determined to be 52% using HPLC analysis on a Chiralcel AD column (hexane/2-propanol = 95/5, 0.5 mL/min, λ = 254 nm); Retention times were 32.9 min (major) and 36.5 min (minor). [α]_D_^20^ +110.8 (*c* 1.00, CHCl_3_). IR (KBr, cm^−1^): 3514(s), 2980(s), 2869(s), 1645(w), 1599(w), 1500(s), 1381(s), 1297(m), 1136(s), 934(m), 845(m), 794(m). ^1^H NMR (400 MHz, CDCl_3_): δ 7.54 (d, *J* = 4.8 Hz, 1H), 7.28–7.23 (m, 4H), 7.13–7.10 (m, 1H), 6.96 (d, *J* = 8.4 Hz, 2H), 6.80 (t, *J* = 7.2 Hz, 1H), 6.58 (d, *J* = 10.0 Hz, 1H), 5.94–5.90 (m, 1H), 5.10 (d, *J* = 9.6 Hz, 1H), 4.73 (dd, *J* = 2.4, 2.4 Hz, 1H), 2.84 (d, *J* = 2.4 Hz, 3H), 2.38 (br s, 1H). ^13^C NMR (100 MHz, CDCl_3_): δ 150.2, 136.5, 131.9, 129.7, 129.2, 128.1, 127.9, 127.7, 126.4, 125.6, 118.1, 114.7, 70.0, 63.5, 33.4. MS (ESI) *m*/*z* Calcd for C_17_H_17_NO (M^+^): 251.13; Found: 251.83. Anal. Calcd for C_17_H_17_NO: C, 81.24; H, 6.82; N, 5.57. Found: C, 81.52; H, 6.92; N, 5.73.

See Supporting Information for details of the syntheses of the new compounds **2b-l**, **3a-j**, and **4a-d**.

## Supporting Information

Experimental procedures and full characterization data for all the new compounds including optical rotations, IR, ^1^H NMR and ^13^C NMR, MS and elemental analysis are provided in the Supporting Information. In addition, X-ray structure data for compound **3f** are given.

File 1 Experimental and analytical data
