# Supplementary material for: Iridium-catalyzed asymmetric ring-opening reactions of oxabicyclic alkenes with secondary amine nucleophiles
Source: Beilstein J Org Chem. 2009 Oct 9;5:53. doi: 10.3762/bjoc.5.53 (PMC2814328; doi:10.3762/bjoc.5.53)

**Iridium-catalyzed asymmetric ring-opening reactions of oxabicyclic alkenes with secondary amine nucleophiles**

Dingqiao Yang,* Ping Hu, Yuhua Long, Yujuan Wu, Heping Zeng, Hui Wang, and Xiongjun Zuo

School of Chemistry and Environment, South China Normal University, Guangzhou 510006, China

*Corresponding author e-mail: [yangdq@scnu.edu.cn](mailto:yangdq@scnu.edu.cn)

**Supporting Information**

**Table of Contents Page**

**Experimental procedures and characterization data S2**

**Crystal structure and data of 3f S26**

**Copies of ^1^H and ^13^C NMR spectra of 2a-l, 3a-j and 4a-d S39**

**Experimental procedures and characterization data**

**General：**All flasks were flame-dried under a stream of nitrogen and cooled before use. Solvents and solutions were transferred with syringes and cannulae using standard inert atmosphere techniques. NMR spectra were recorded at 400 MHz using a Varian INOVA NMR spectrometer with CDCl_3_ as reference standard (δ 7.27 ppm) for ^1^H NMR and (δ 77.23 ppm) for ^13^C NMR. Spectral features are tabulated in the following order: Chemical shift (δ, ppm); number of protons; multiplicity (s-singlet, d-doublet, t-triplet, m-complex multiplet and br-broad); coupling constants (*J*, Hz). IR spectra were obtained using a Nicolet DX FT-IR spectrometer as a KBr pellet or using a Perkin-Elmer Spectrum 1000 FT-IR spectrometer as a neat film on a NaCl plate. MS spectra were recorded on a Bruker esquire 6000 mass spectrometer (ESI). Optical rotations were measured on a Perkin-Elmer Model 243 Polarimeter using the sodium D line with spectra-grade CHCl_3_ in a 1 dm cell. Melting points were taken on an XT_4_ binocular micro melting point apparatus. HPLC analysis was performed on an Agilent 1100 Series HPLC with a Chiralcel AD column. Elemental analysis was conducted on a Thermo. Flash EA.TM.1112. Crystal structure determination was carried out on a Bruker SMART-1000 X-ray diffraction apparatus.

**Materials:** DME was distilled from sodium benzophenone ketyl and stored. THP, THF, dioxane, and toluene were distilled from sodium benzophenone ketyl immediately prior to use. CH_3_CN was distilled from calcium hydride. DMF was dried over MgSO_4_ and stored over activated molecular sieves.

**Preparation of di-*μ*-chlorobis(1,5-cyclooctadiene)diiridium (I)**

To a 150.0 mL 3-neck flask was added IrCl_3_·3H_2_O (1.0 g, 3.0 mmol) followed by addition of 95% of ethanol (17.0 mL), water (8.5 mL) and 1,5-cyclooctadiene (3.0 mL, 24.5 mmol) under nitrogen atmosphere. The resulting mixture was stirred under reflux for 24 h and formed a brick red precipitate. It was allowed to cool to room temperature and filtered. The precipitate was rinsed with cold methanol to remove the trace amount of 1,5-cyclooctadiene unreacted. Then the product was dried in vacuum for 8 h. And [Ir(COD)Cl]_2_ was obtained in 67% yield as a brick red solid. mp 200–202 °C.

**Preparation of 1,4-dihydro-1,4-epoxynaphthalene (1a)**

To a 100 mL round bottom flask with straight condenser tube 10 mL furan and 10 mL DME were added. Taking two 25 mL dropping bottles, one with 4 mL isoamyl nitrite and 10 mL DME (A), the other with 2.75 g (20.06 mmol) 2-aminobenzoic acid dissolved by 10 mL DME (B). Then 1 mL A and 1 mL B were added to the refluxing furan solution per 4 min. Firstly the A was added, then the B. The solution became red brown, giving off gas when the reagents were added. Let the mixture refluxing till the solution did not release gas after all the reactants were added (about 15 min). After completion 25 mL 2% sodium hydroxide was added to the mixture and transferred to separating funnel to rinse, which we can get the organic phase and the aqueous solution extracted three times by 15 mL petroleum ether (bp 30–60 °C). Then the extractive solution and the organic phase were mixed together. The mixture was washed by water (15 mL × 4) and dried by anhydrous magnesium sulfate. After completion the reaction mixture was concentrated in vacuo and the solvents were removed, the crude mixture was purified by flash chromatography gave **1a** a yellow solid (1.72 g, 60%). *R_f_* = 0.45 on silica gel (25% ethyl acetate in petroleum ether). mp 55–56 °C. IR (thin film, cm^–1^): 3125(s), 3040(s), 3020(s), 1958(s), 1916(s), 1814(s), 1620(s), 1562(s), 1449(s), 1345(s), 1278(s), 1195(s), 1164(s), 1128(s), 1073(s), 986(s), 938(s), 844(s), 765(s), 689(s), 635(s). MS (ESI) *m*/*z* Calcd for C_10_H_8_O (M^+^): 144.06; Found: 145.06.

**Preparation of 1,4-dihydro-5,8-dimethoxy-1,4-epoxynaphthalene (1b)**

A stirred solution of 2-chloro-1,4-dimethoxy-benzene (1.208 g, 7.0 mmol) in anhydrous THF (12.5 mL), furan (15 mL) was cooled to -78 °C under N_2_ and was treated dropwise with *n*-butyllithium (1.29 mol/L) in hexane (5.3 mL). The solution was stirred at -78 °C for 1 h and added 20 mL distilled water to warm up to room temperature. The reaction mixture was extracted from diethyl ether and dried over Na_2_SO_4_. The ether was then removed in vacuo, the crude mixture was purified by flash chromatography (15% ethyl acetate in petroleum ether) gave **1b** a white solid (0.89 g, 63%). *R_f_* = 0.21 on silica gel (15% ethyl acetate in petroleum ether); mp 147–149 °C. MS (ESI) *m*/*z* Calcd for C_12_H_12_O_3_ (M^+^): 204.22; Found: 205.20. IR (thin film, cm^–1^): 2960(m), 2934(m), 1736(m), 1598(m), 1467(s), 1326(s), 1287(s), 1063(s). ^1^H NMR (400 MHz, CDCl_3_): δ 7.01 (2H, s), 6.94 (2H, s), 5.64 (2H, s), 3.81 (6H, s). ^13^C NMR (100 MHz, CDCl_3_): δ 145.8, 143.3, 141.7, 106.8, 82.6, 56.5.

**Preparation of 1,4-dihydro-6,7-dimethoxy-1,4-epoxynaphthalene (1c)**

A solution of LDA was freshly prepared by addition of 7.4 mL (1.1 equiv) in anhydrous THF (15 mL) at -78 °C, followed by a brief warm-up to room temperature. Furan (15 mL) was slowly added at -78 °C and the resulting colorless solution was treated dropwise with a solution of 1-bromo-2,5-dimethoxy-benzene (2.16 g, 10 mmol) in anhydrous THF (5 mL). The yellow solution was kept at -78 °C for 30 min, then quenched with water and left to warm up. Standard extractive work-up with diethyl ether and brine yielded the crude product which was purified by flash chromatography (50% ethyl acetate in hexanes) to give **1c** a white solid (2.10 g, 86%). *R_f_* = 0.17 on silica gel (50% ethyl acetate in hexanes). mp 86–87 °C (Et_2_O). IR (KBr, cm^–1^): 3085(s), 2989(s), 2839(s), 1619(m), 1438(s), 1253(s), 1139(s), 1075(s), 997(s). ^1^H NMR (400 MHz, CDCl_3_): δ 7.06 (2H, s), 6.97 (2H, s), 6.54 (2H, s), 5.92 (2H, s), 3.78 (6H, s). ^13^C NMR (100 MHz, CDCl_3_): δ 148.1, 143.1, 111.9, 80.6, 56.6.

**General Procedure (I) for the asymmetric ring-opening reactions of oxabenzonorbornadiene 1a with substituted *N*-alkylaniline:** A 5.0 mL round bottom flask fitted with a reflux condenser was flame-dried under a stream of nitrogen and cooled to room temperature. [Ir(COD)Cl]_2_ (5.8 mg, 2.5 mol %) and (*S*)-*p*-Tol-BINAP (10.7 mg, 5 mol %) were simultaneously added and followed by addition of anhydrous tetrahydrofuran (2.0 mL). After they were stirred for about 10 min, oxabenzonorbornadiene **1a** (50.0 mg, 0.347 mmol) was added and the resulting mixture was heated to reflux. On the first sign of reflux, nucleophile (3.0 equiv to **1a**) was added. Then the temperature was continuously increased to 80 °C until the reaction was completed as judged by thin layer chromatography. The reaction mixture was then concentrated in vacuo and purified by column chromatography (silica gel: 200–300 mesh) to give the target product.

**General Procedure (II) for the asymmetric ring-opening reactions of Oxabenzonorbornadiene 1a with aliphatic secondary amines**: A 5.0 mL round bottom flask fitted with a reflux condenser was flame-dried under a stream of nitrogen and cooled to room temperature. [Ir(COD)Cl]_2_ (5.8 mg, 2.5 mol %) and (*S*)-*p*-Tol-BINAP (10.7 mg, 5 mol %) were simultaneously added and followed by addition of anhydrous tetrahydrofuran (2.0 mL). After they were stirred for about 5 min, NH_4_I (50 mg, 1 equiv to **1a**) was added. After they were stirred for about 10 min, oxabenzonorbornadiene **1a** (50.0 mg, 0.347 mmol) was added and the resulting mixture was heated to reflux. On the first sign of reflux, aliphatic secondary amine (3 equiv to **1a**) was added. The temperature was then continuously increased to 80 °C until the reaction was completed as judged by thin layer chromatography. The reaction mixture was then concentrated in vacuo and purified by column chromatography (silica gel: 200–300 mesh) to give the target product.

**General Procedure (III) for the asymmetric ring-opening reactions of substituted oxabenzonorbornadiene 1b with substituted *N*-alkylaniline:** A 5.0 mL round bottom flask fitted with a reflux condenser was flame-dried under a stream of nitrogen and cooled to room temperature. [Ir(COD)Cl]_2_ (5.8 mg, 2.5 mol %) and (*S*)-*p*-Tol-BINAP (10.7 mg, 5 mol %) were simultaneously added and followed by addition of anhydrous tetrahydrofuran (2.0 mL). After they were stirred for about 5 min, NH_4_I (50 mg, 1 equiv to **1a**) was added. After they were stirred for about 10 min, substituted oxabenzonorbornadiene **1b** (71.0 mg, 0.347 mmol) was added and the resulting mixture was heated to reflux. On the first sign of reflux, substituted *N*-alkylaniline (3 equiv to **1b**) was added. Then the temperature was continuously increased to 100 °C until the reaction was completed as judged by thin layer chromatography. The reaction mixture was then concentrated in vacuo and purified by column chromatography (silica gel: 200–300 mesh) to give the target product.

**General Procedure (IV) for the asymmetric ring-opening reactions of substituted oxabenzonorbornadiene 1b with aliphatic secondary amines:** A 5.0 mL round bottom flask fitted with a reflux condenser was flame-dried under a stream of nitrogen and cooled to room temperature. [Ir(COD)Cl]_2_ (5.8 mg, 2.5 mol %) and (*S*)-*p*-Tol-BINAP (10.7 mg, 5 mol %) were simultaneously added and followed by addition of anhydrous tetrahydrofuran (2.0 mL). After they were stirred for about 5 min, NH_4_I (50.0 mg, 1 equiv to **1b**) was added. After they were stirred for about 10 min, substituted oxabenzonorbornadiene **1b** (71.0 mg, 0.347 mmol) was added and the resulting mixture was heated to reflux. On the first sign of reflux, substituted aliphatic secondary amine (3 equiv to **1b**) was added. Then the temperature was continuously increased to 100 °C until the reaction was completed as judged by thin layer chromatography. The reaction mixture was then concentrated in vacuo and purified by column chromatography (silica gel: 200–300 mesh) to give the target product.

**(1*S*,2*S*)-2-[Methyl(phenyl)amino]-1,2-dihydronaphthalen-1-ol (2a)**

Following the general procedure (**I**), **2a** was obtained as colorless oil (76.0 mg, 87%). *R_f_* = 0.17 on silica gel (ethyl acetate:petroleum ether = 1:20, v/v). The ee was determined to be 52% using HPLC analysis on a Chiralcel AD column (hexane/2-propanol = 95/5, 0.5 mL/min, λ = 254 nm); Retention times were 32.9 min (major) and 36.5 min (minor). [α]_D_^20^ +110.8 (*c* 1.00, CHCl_3_). IR (KBr, cm^–1^): 3514(s), 2980(s), 2869(s), 1645(w), 1599(w), 1500(s), 1381(s), 1297(m), 1136(s), 934(m), 845(m), 794(m). ^1^H NMR (400 MHz, CDCl_3_): δ 7.54 (d, *J* = 4.8 Hz, 1H), 7.28–7.23 (m, 4H), 7.13–7.10 (m, 1H), 6.96 (d, *J* = 8.4 Hz, 2H), 6.80 (t, *J* = 7.2 Hz, 1H), 6.58 (d, *J* = 10.0 Hz, 1H), 5.94–5.90 (m, 1H), 5.10 (d, *J* = 9.6 Hz, 1H), 4.73 (dd, *J* = 2.4, 2.4 Hz, 1H), 2.84 (d, *J* = 2.4 Hz, 3H), 2.38 (br s, 1H). ^13^C NMR (100 MHz, CDCl_3_): δ 150.2, 136.5, 131.9, 129.7, 129.2, 128.1, 127.9, 127.7, 126.4, 125.6, 118.1, 114.7, 70.0, 63.5, 33.4. MS (ESI) *m*/*z* Calcd for C_17_H_17_NO (M^+^): 251.13; Found: 251.83. Anal. Calcd for C_17_H_17_NO: C, 81.24; H, 6.82; N, 5.57. Found: C, 81.52; H, 6.92; N, 5.73.

**(1*S*,2*S*)-2-[Ethyl(phenyl)amino]-1,2-dihydronaphthalen-1-ol (2b)**

Following the general procedure (**I**), **2b** was obtained as colorless oil (69.0 mg, 75%). *R_f_* = 0.15 on silica gel (ethyl acetate:petroleum ether = 1:20, v/v). The ee was determined to be 56% using HPLC analysis on a Chiralcel AD column (hexane/2-propanol = 95/5, 0.5 mL/min, λ = 254 nm); Retention times were 27.0 min (major) and 28.6 min (minor). [α]_D_^20^ +113.2 (*c* 1.00, CHCl_3_). IR (KBr, cm^–1^): 3434(m), 3057(w), 3031(w), 2928(s), 2843(m), 1645(w), 1597(s), 1504(s), 1375(m), 1255(m), 989(m). ^1^H NMR (400 MHz, CDCl_3_): δ 7.5 (br s, 1H), 7.26–7.21 (m, 4H), 7.12–7.05 (m, 1H), 7.00 (dd, *J* = 4.8, 2.8 Hz, 2H), 6.77 (br s, 1H), 6.58 (d, *J* = 8.4 Hz, 1H), 5.95–5.92 (m, 1H), 5.1 (s, 1H), 4.6 (s, 1H), 3.35 (s, 2H), 2.33 (br s, 1H), 1.50 (s, 3H). ^13^C NMR (100 MHz, CDCl_3_): δ 148.3, 136.4, 132.0, 129.5, 129.3, 128.7, 128.1, 126.6, 126.0, 117.7, 114.8, 112.8, 70.3, 63.3, 41.6, 14.5. MS (ESI) *m*/*z* Calcd for C_18_H_19_NO (M^+^): 265.15; Found: 266.02 (M+H)^+^. Anal. Calcd for C_18_H_19_NO: C, 81.47; H, 7.22; N, 5.28. Found: C, 81.71; H, 7.43; N, 5.41.

**(1*S*,2*S*)-2-[Allyl(phenyl)amino]-1,2-dihydronaphthalen-1-ol (2c)**

Following the general procedure (**I**), **2c** was obtained as colorless oil (46.0 mg, 48%). *R_f_* = 0.18 on silica gel (ethyl acetate:petroleum ether = 1:20, v/v). The ee was determined to be 74% using HPLC analysis on a Chiralcel AD column (hexane/2-propanol = 95/5, 0.5 mL/min, λ = 254 nm); Retention times were 29.7 min (major) and 31.4 min (minor). [α]_D_^20^ +153.4 (*c* 1.00, CHCl_3_). IR (KBr, cm^–1^): 3418(s), 3055(w), 3048(w), 2928(w), 2851(s), 2353(w), 1642(m), 1600(s), 1481(s), 1161(w), 974(w), 923(w). ^1^H NMR (400 MHz, CDCl_3_): δ 7.52 (d, *J* = 3.6 Hz, 1H), 7.30–7.22 (m, 4H), 7.14 (t, *J* = 4.0 Hz, 1H), 6.95–6.93 (m, 2H), 6.80–6.76 (m, 1H), 6.63 (br d, *J* = 9.6 Hz, 1H), 5.97–5.93 (m, 2H), 5.25–5.09 (m, 3H), 4.77 (br s, 1H), 3.91 (d, *J* = 11.0 Hz, 2H), 2.31 (s, 1H). ^13^C NMR (100 MHz, CDCl_3_): δ 148.7, 136.3, 136.0, 131.9, 129.9, 129.2, 128.2, 128.1, 126.6, 126.1, 119.3, 117.7, 116.0, 114.4, 70.3, 62.3, 50.1. MS (ESI) *m*/*z* Calcd for C_19_H_19_NO (M^+^): 277.15; Found: 277.97. Anal. Calcd for C_19_H_19_NO: C, 82.28; H, 6.90; N, 5.05. Found: C, 82.41; H, 7.11; N, 4.86.

**(1*S*,2*S*)-2-[(3-Chlorophenyl)(methyl)amino]-1,2-dihydronaphthalen-1-ol (2d)**

Following the general procedure (**I**), **2d** was obtained as colorless oil (56.0 mg, 56%). *R_f_* = 0.12 on silica gel (ethyl acetate:petroleum ether = 1:20, v/v). The ee was determined to be 72% using HPLC analysis on a Chiralcel AD column (hexane/2-propanol = 95/5, 0.5 mL/min, λ = 254 nm); Retention times were 38.6 min (major) and 45.3 min (minor). [α]_D_^20^ +151.8 (*c* 1.00, CHCl_3_). IR (KBr, cm^–1^): 3426(m), 2935(m), 2919(m), 1600(w), 1597(s), 1495(s), 1358(w), 1100(m), 972(m). ^1^H NMR (400 MHz, CDCl_3_): δ 7.54 (d, *J* = 3.6 Hz, 1H), 7.32–7.26 (m, 2H), 7.17–7.12 (m, 2H), 6.92–6.74 (m, 3H), 6.62 (dd, *J* = 2.4, 2.4 Hz, 1H), 5.89 (dd, *J* = 3.2, 2.8 Hz, 1H), 5.07 (d, *J* = 9.2 Hz, 1H), 4.75–4.69 (m, 1H), 2.83 (s, 3H), 1.26 (s, 1H). ^13^C NMR (100 MHz, CDCl_3_): δ 151.3, 136.3, 135.2, 131.8, 130.1, 130.0, 128.3, 128.1, 127.2, 126.6, 125.8, 117.7, 114.2, 112.4, 70.2, 63.2, 33.4. MS (ESI) *m*/*z* Calcd for C_17_H_16_ClNO (M^+^): 285.09; Found: 285.91. Anal. Calcd for C_17_H_16_ClNO: C, 71.45; H, 5.64; N, 4.90. Found: C, 71.52; H, 5.48; N, 4.73.

**(1*S*,2*S*)-2-[(4-Chlorophenyl)(methyl)amino]-1,2-dihydronaphthalen-1-ol (2e)**

Following the general procedure (**I**), **2e** was obtained as a white solid (70.0 mg, 70%). *R_f_* = 0.16 on silica gel (ethyl acetate:petroleum ether = 1:20, v/v). mp 84–85 °C. The ee was determined to be 55% using HPLC analysis on a Chiralcel AD column (hexane/2-propanol = 95/5, 0.5 mL/min, λ = 254 nm); Retention times were 44.0 min (minor) and 51.2 min (major). [α]_D_^20^ +112.8 (*c* 1.00, CHCl_3_). IR (KBr, cm^–1^): 3417(m), 3038(m), 2887(m), 1645(w), 1586(m), 1493(s), 1372(w), 1024(m), 944(w), 813(m). ^1^H NMR (400 MHz, CDCl_3_): δ 7.52 (t, *J* = 4.8 Hz, 1H), 7.28–7.10 (m, 5H), 6.86 (d, *J* = 8.8 Hz, 2H), 6.59 (d, *J* = 9.6 Hz, 1H), 5.89 (dd, *J* = 3.2, 2.8 Hz, 1H), 5.06 (dd, *J* = 3.2, 3.2 Hz, 1H), 4.67–4.64 (m, 1H), 2.80 (s, 3H), 2.22 (br s, 1H). ^13^C NMR (100 MHz, CDCl_3_): δ 148.7, 136.3, 131.8, 129.9, 129.0, 128.2, 128.1, 127.2, 126.6, 125.7, 122.9, 115.7, 70.1, 63.6, 33.5. MS (ESI) *m*/*z* Calcd for C_17_H_16_ClNO (M^+^): 285.09; Found: 285.60. Anal. Calcd for C_17_H_16_ClNO: C, 71.45; H, 5.64; N, 4.90. Found: C, 71.45; H, 5.31; N, 4.90.

**(1*S*,2*S*)-2-[(4-Fluorophenyl)(methyl)amino]-1,2-dihydronaphthalen-1-ol (2f)**

Following the general procedure (**I**), **2f** was obtained as a white solid (61.0 mg, 65%). *R_f_* = 0.17 on silica gel (ethyl acetate:petroleum ether = 1:20, v/v). mp 76–78 °C. The ee was determined to be 51% using HPLC analysis on a Chiralcel AD column (hexane/2-propanol = 95/5, 0.5 mL/min, λ = 254 nm); Retention times were 42.0 min (major) and 39.7 min (minor). [α]_D_^20^ +108.4 (*c* 1.00, CHCl_3_). IR (KBr, cm^–1^): 3425(m), 3051(w), 2892(m), 2817(m), 1600(m), 1500(s), 1357(w), 1225(s), 1108(m), 933(m), 824(s). ^1^H NMR (400 MHz, CDCl_3_): δ 7.55 (t, *J* = 3.2 Hz, 1H), 7.27–7.24 (m, 2H), 7.09 (t, *J* = 6.0 Hz, 1H), 6.97–6.88 (m, 4H), 6.58 (dd, *J* = 2.4, 2.4 Hz, 1H), 5.90 (dd, *J* = 2.8, 2.8 Hz, 1H), 5.07 (br d, *J* = 7.6 Hz, 1H), 4.59–4.55 (m, 1H), 2.79 (s, 3H), 2.41 (br s, 1H). ^13^C NMR (100 MHz, CDCl_3_): δ 157.5, 155.1, 146.9, 136.5, 131.9, 129.8, 128.1, 127.9, 127.2, 126.5, 116.5, 115.5, 69.9, 64.7, 33.8. MS (ESI) *m*/*z* Calcd for C_17_H_16_FNO (M^+^): 269.12; Found: 269.94. Anal. Calcd for C_17_H_16_FNO: C, 75.82; H, 5.99; N, 5.20. Found: C, 75.84; H, 5.96; N, 5.17.

**(1*S*,2*S*)-2-[(4-Bromophenyl)(methyl)amino]-1,2-dihydronaphthalen-1-ol (2g)**

Following the general procedure (**I**), **2g** was obtained as a white solid (81.0 mg, 71%). *R_f_* = 014 on silica gel (ethyl acetate:petroleum ether = 1:15, v/v). mp 89–90 °C. The ee was determined to be 65% using HPLC analysis on a Chiralcel AD column (hexane/2-propanol = 95/5, 0.5 mL/min, λ = 254 nm); Retention times were 57.4 min (major) and 48.4 min (minor). [α]_D_^20^ +152.3 (*c* 1.00, CHCl_3_). IR (KBr, cm^–1^): 3424(m), 3038(w), 2887(m), 2883(m), 2341(w), 1720(w), 1591(m), 1493(s), 1372(m), 1099(m), 812(m). ^1^H NMR (400 MHz, CDCl_3_): δ 7.51 (t, *J* = 3.6 Hz, 1H), 7.31–7.24 (m, 4H), 7.10 (t, *J* = 5.6 Hz, 1H), 6.80 (d, *J* = 8.8 Hz, 2H), 6.59 (dd, *J* = 2.4, 2.4 Hz, 1H), 5.87 (dd, *J* = 2.8, 3.2 Hz, 1H), 5.05 (d, *J* = 9.6 Hz, 1H), 4.67–4.63 (m, 1H), 2.79 (s, 3H), 2.58 (br s, 1H). ^13^C NMR (100 MHz, CDCl_3_): δ 149.1, 136.2, 131.9, 131.8, 129.9, 128.2, 128.1, 127.2, 126.6, 125.7, 116.1, 109.9, 70.1, 63.4, 33.5. MS (ESI) *m*/*z* Calcd for C_17_H_16_BrNO (M^+^): 329.04; Found: 330.01 (M+H)^+^. Anal. Calcd for C_17_H_16_BrNO: C, 61.83; H, 4.88; N, 4.24. Found: C, 61.76; H, 4.79; N, 4.23.

**(1*S*,2*S*)-2-[(4-Methoxyphenyl)(methyl)amino]-1,2-dihydronaphthalen-1-ol (2h)**

Following the general procedure (**I**), **2h** was obtained as colorless oil (83.0 mg, 85%). *R_f_* = 0.13 on silica gel (ethyl acetate:petroleum ether = 1:20, v/v). The ee was determined to be 50% using HPLC analysis on a Chiralcel AD column (hexane/2-propanol = 95/5, 0.5 mL/min, λ = 254 nm); Retention times were 36.6 min (major) and 44.1 min (minor). [α]_D_^20^ +107.8 (*c* 1.00, CHCl_3_). IR (KBr, cm^–1^): 3441(m), 3021(m), 2915(m), 2827(m), 2333(w), 1629(m), 1500(m), 1459(m), 1046(m), 933(w), 779(s). ^1^H NMR (400 MHz, CDCl_3_): δ 7.56 (t, *J* = 6.4 Hz, 1H), 7.29–7.21 (m, 2H), 7.07 (dd, *J* = 2.4, 2.4 Hz, 1H), 6.95–6.92 (m, 2H), 6.85–6.80 (m, 2H), 6.54 (dd, *J* = 2.8, 2.4 Hz, 1H), 5.93 (dd, *J* = 2.8, 2.8 Hz, 1H), 5.07 (d, *J* = 10 Hz, 1H), 4.53–4.49 (m, 1H), 3.75 (s, 3H), 2.78 (s, 3H), 1.24 (br s, 1H). ^13^C NMR (100 MHz, CDCl_3_): δ 153.0, 144.6, 136.6, 131.9, 129.5, 128.0, 127.7, 127.3, 126.3, 125.3, 117.5, 114.6, 69.6, 65.3, 55.6, 33.9. MS (ESI) *m*/*z* Calcd for C_18_H_19_NO_2_ (M^+^): 281.14; Found: 281.18. Anal. Calcd for C_18_H_19_NO_2_: C, 76.84; H, 6.81; N, 4.98. Found: C, 76.90; H, 6.72; N, 4.69.

**(1*S*,2*S*)-2-(Piperidin-1-yl)-1,2-dihydronaphthalen-1-ol (2i)**

Following the general procedure (**II**), **2i** was obtained as colorless oil (72.0 mg, 90%). *R_f_* = 0.2 on silica gel (ethyl acetate:petroleum ether = 1:4, v/v). The ee was determined to be 49% using HPLC analysis on a Chiralcel AD column (hexane/2-propanol = 90/10, 1.0 mL/min, λ = 254 nm); Retention times were 4.9 min (major) and 4.6 min (minor). [α]_D_^20^ +102.6 (*c* 1.00, CHCl_3_). IR (KBr, cm^–1^): 3317(m), 3052(w), 2931(s), 2854(m), 1630(w), 1577(m), 1500(w), 1455(m), 1386(m), 1279(m), 773(m). ^1^H NMR (400 MHz, CDCl_3_): δ 7.56 (d, *J* = 7.2 Hz, 1H), 7.20–7.17 (m, 2H), 7.04 (d, *J* = 7.2 Hz, 1H), 6.48 (d, *J* = 10 Hz, 1H), 6.10 (d, *J* = 9.6 Hz, 1H), 4.86 (br d, *J* = 12 Hz, 1H), 3.37 (d, *J* = 6.0 Hz, 2H), 2.80–2.73 (m, 2H), 2.46–2.42 (m, 2H), 1.63–1.46 (m, 6H). ^13^C NMR (100 MHz, CDCl_3_): δ 137.4, 131.8, 128.8, 127.6, 127.1, 125.9, 125.1, 124.5, 68.2, 67.6, 50.4, 26.5, 24.6. MS (ESI) *m*/*z* Calcd for C_15_H_19_NO (M^+^): 229.15; Found: 230.19 (M+H)^+^. Anal. Calcd for C_15_H_19_NO: C, 78.56; H, 8.35; N, 6.11. Found: C, 78.44; H, 8.38; N, 6.04.

**(1*S*,2*S*)-2-(3,4-Dihydro-1*H*-isoquinolin-2-yl)-1,2-dihydronaphthalen-1-ol (2j)**

Following the general procedure (**II**), **2j** was obtained as colorless oil (70.0 mg, 72%). *R_f_* = 0.37 on silica gel (ethyl acetate:petroleum ether = 1:6, v/v). mp 179–180 °C. The ee was determined to be 65% using HPLC analysis on a Chiralcel AD column (hexane/2-propanol = 95/5, 0.5 mL/min, λ = 254 nm); Retention times were 30.3 min (major) and 31.6 min (minor). [α]_D_^20^ +150.4 (*c* 1.00, CHCl_3_). IR (KBr, cm^–1^): 3441(m), 3021(m), 2915(s), 2827(s), 2333(w), 1629(m), 1500(m), 1459(s), 1046(m), 933(m), 779(s), 747(s). ^1^H NMR (400 MHz, CDCl_3_): δ 7.59 (d, *J* = 7.6 Hz, 1H), 7.29–7.21 (m, 2H), 7.14–7.02 (m, 5H), 6.57 (dd, *J* = 2.4, 2.4 Hz, 1H), 6.14 (dd, *J* = 2.0, 2.0 Hz, 1H), 5.01 (d, *J* = 12.0 Hz, 1H), 4.02 (br d, *J* = 15.2 Hz, 1H), 3.79 (br d, *J* = 15.2 Hz, 1H), 3.64 (br d, *J* = 12.0 Hz, 1H), 3.16–3.11 (m, 1H), 2.95 (t, *J* = 6.0 Hz, 2H), 2.82–2.76 (m, 1H), 2.16 (s, 1H). ^13^C NMR (100 MHz, CDCl_3_): δ 137.0, 134.7, 134.4, 131.7, 129.6, 128.8, 127.9, 127.4, 126.5, 126.2, 126.1, 125.7, 124.6, 124.5, 68.0, 67.6, 52.0, 46.9, 29.9. MS (ESI) *m*/*z* Calcd for C_19_H_19_NO (M^+^): 277.15; Found: 278.15 (M+H)^+^. Anal. Calcd for C_19_H_19_NO: C, 82.28; H, 6.90; N, 5.05. Found: C, 82.19; H, 6.79; N, 4.75.

**(1*S*,2*S*)-2-(4-Phenylpiperazin-1-yl)-1,2-dihydronaphthalen-1-ol (2k)**

Following the general procedure (**II**), **2k** was obtained as a white solid (93.0 mg, 88%). *R_f_* = 0.42 on silica gel (ethyl acetate:petroleum ether = 1:1, v/v). mp 155–156 °C. The ee was determined to be 53% using HPLC analysis on a Chiralcel AD column (hexane/2-propanol = 98/2, 0.5 mL/min, λ = 254 nm); Retention times were 35.9 min (major) and 31.2 min (minor). [α]_D_^20^ +111.6 (*c* 1.00, CHCl_3_). IR (KBr, cm^–1^): 3345(m), 3016(w), 2928(s), 2824(s), 1597(m), 1492(s), 1452(s), 1369(m), 1226(s), 1169(s), 1133(m), 1045(m), 763(s). ^1^H NMR (400 MHz, CDCl_3_): δ 7.60 (d, *J* = 7.2 Hz, 1H), 7.30–7.23 (m, 4H), 7.10 (d, *J* = 8.4 Hz, 1H), 6.94 (d, *J* = 8.0 Hz, 2H), 6.89 (t, *J* = 7.2 Hz, 1H), 6.57 (dd, *J* = 2.4, 2.4 Hz, 1H), 6.13 (dd, *J* = 2.4, 2.4 Hz, 1H), 4.93 (d, *J* = 11.6 Hz, 1H), 3.53 (br d, *J* = 11.6 Hz, 1H), 3.44 (br s, 1H), 3.24–3.17 (m, 4H), 2.99–2.94 (m, 2H), 2.74–2.69 (m, 2H). ^13^C NMR (100 MHz, CDCl_3_): δ 151.4, 137.2, 131.9, 129.6, 129.3, 128.1, 127.6, 126.4, 125.0, 124.5, 120.1, 116.4, 67.9, 67.6, 49.9, 49.1. MS (ESI) *m*/*z* Calcd for C_20_H_22_N_2_O (M^+^): 306.17; Found: 307.16 (M+H)^+^. Anal. Calcd for C_20_H_22_N_2_O: C, 78.40; H, 7.24; N, 9.14. Found: C, 78.21; H, 7.31; N, 9.26.

**(1*S*,2*S*)-2-[4-(3,4-Dichlorophenyl)piperazin-1-yl]-1,2-dihydronaphthalen-1-ol (2l)**

Following the general procedure (**II**), **2l** was obtained as a white solid (101.2 mg, 78%). *R_f_* = 0.2 on silica gel (ethyl acetate:petroleum ether = 1:4, v/v). mp 138–139 °C. The ee was determined to be 56% using HPLC analysis on a Chiralcel AD column (hexane/2-propanol = 90/10, 0.5 mL/min, λ = 254 nm); Retention times were 38.2 min (major) and 40.4 min (minor). [α]_D_^20^ +100.3 (*c* 1.00, CHCl_3_). IR (KBr, cm^–1^): 3357(m), 3028(w), 2924(m), 2835(m), 1592(s), 1552(w), 1482(s), 1451(s), 1235(s), 1138(m), 955(m), 781(s). ^1^H NMR (400 MHz, CDCl_3_): δ 7.56 (d, *J* = 7.2 Hz, 1H), 7.28–7.21 (m, 3H), 7.07 (br d, *J* = 4.0 Hz, 1H), 6.95 (d, *J* = 2.8 Hz, 1H), 6.73 (dd, *J* = 2.8, 2.8 Hz, 1H), 6.55 (dd, *J* = 2.4, 2.4 Hz, 1H), 6.08 (dd, *J* = 2.4, 2.8 Hz, 1H), 4.90 (d, *J* = 11.2 Hz, 1H), 3.51 (d, *J* = 11.2 Hz, 1H), 3.21–3.17 (m, 5H), 2.95–2.92 (m, 2H), 2.71–2.67 (m, 2H). ^13^C NMR (100 MHz, CDCl_3_): δ 150.6, 136.8, 132.8, 131.6, 130.4, 129.6, 128.0, 127.6, 126.3, 124.8, 124.0, 122.4, 117.4, 115.4, 67.8, 67.4, 49.3, 48.7. MS (ESI) *m*/*z* Calcd for C_20_H_20_Cl_2_N_2_O (M^+^): 374.10; Found: 375.13 (M+H)^+^. Anal. Calcd for C_20_H_20_Cl_2_N_2_O: C, 64.01; H, 5.37; N, 7.46. Found: C, 64.22; H, 5.41; N, 7.47.

**(1*S*,2*S*)-5,8-Dimethoxy-2-(methyl(phenyl)amino)-1,2-dihydronaphthalen-1-ol (3a)**

Following the general procedure (**III**), **3a** was obtained as a white solid (87.0 mg, 81%). *R_f_* = 0.08 on silica gel (ethyl acetate:petroleum ether = 1:8, v/v). mp 110–111 °C. The ee was determined to be 76% using HPLC analysis on a Chiralcel AD column (hexane/2-propanol = 90/10, 0.5 mL/min, λ = 254 nm); Retention times were 40.5 min (major) and 45.0 min (minor). [α]_D_^20^ +117.5 (*c* 1.00, CHCl_3_). IR (KBr, cm^–1^): 3439(m), 2936(m), 2828(m), 2381(w), 2318(w), 1593(s), 1484(s), 1268(s), 1114(m), 1013(m), 797(w). ^1^H NMR (400 MHz, CDCl_3_): δ 7.27 (t, *J* = 10 Hz, 2H), 7.16 (dd, *J* = 1.6, 1.6 Hz, 1H), 6.97 (br d, *J* = 8.0 Hz, 2H), 6.82–6.75 (m, 3H), 5.97–5.94 (m, 1H), 5.17 (br s, 1H), 4.69 (br s, 1H), 3.83 (br s, 3H), 3.79 (br s, 3H), 2.55 (s, 3H), 2.42 (br s, 1H). ^13^C NMR (100 MHz, CDCl_3_): δ 151.0, 149.8, 129.2, 124.6, 123.1, 117.2, 117.2, 113.8, 113.7, 111.3, 110.5, 64.8, 58.7, 58.7, 56.2, 55.8, 32.6. MS (ESI) *m*/*z* Calcd for C_19_H_21_NO_3_ (M^+^): 311.15; Found: 334.2 (M+Na)^+^. Anal. Calcd for C_19_H_21_NO_3_: C, 73.29; H, 6.80; N, 4.50. Found: C, 73.29; H, 6.80; N, 4.50.

**(1*S*,2*S*)-2-[Ethyl(phenyl)amino]-5,8-dimethoxy-1,2-dihydronaphthalen-1-ol (3b)**

Following the general procedure (**III**), **3b** was obtained as a white solid (68.0 mg, 70%). *R_f_* = 0.10 on silica gel (ethyl acetate:petroleum ether = 1:8, v/v). mp 143–144 °C. The ee was determined to be 81% using HPLC analysis on a Chiralcel AD column (hexane/2-propanol = 90/10, 0.5 mL/min, λ = 254 nm); Retention times were 35.6 min (major) and 37.7 min (minor). [α]_D_^20^ +120.8 (*c* 1.00, CHCl_3_). IR (KBr, cm^–1^): 3421(m), 2936(m), 2828(m), 2338(w), 1652(m), 1589(m), 1501(m), 1262(m), 1095(w), 800(w), 753(w). ^1^H NMR (400 MHz, CDCl_3_): δ 7.22–7.16 (m, 2H), 7.12–7.08 (m, 1H), 6.88–6.85 (m, 2H), 6.76–6.65 (m, 3H), 5.96–5.91 (m, 1H), 5.10 (s, 1H), 4.57 (s, 1H), 3.76 (s, 3H), 3.70 (s, 3H), 3.02–2.99 (m, 2H), 2.36 (t, *J* = 4.0 Hz, 1H), 0.90–0.87 (m, 3H). ^13^C NMR (100 MHz, CDCl_3_): δ 150.9, 149.5, 147.3, 129.0, 125.3, 124.2, 122.6, 121.5, 116.0, 112.9, 111.0, 110.2, 64.4, 57.8, 55.9, 55.5, 39.9, 14.2. MS (ESI) *m*/*z* Calcd for C_20_H_23_NO_3_ (M^+^): 325.17; Found: 326.22 (M+H)^+^. Anal. Calcd for C_20_H_23_NO_3_: C, 73.82; H, 7.12; N, 4.30. Found: C, 73.64; H, 7.42; N, 4.68.

**(1*S*,2*S*)-5,8-Dimethoxy-2-[methyl(*m*-tolyl)amino]-1,2-dihydronaphthalen-1-ol (3c)**

Following the general procedure (**III**), **3c** was obtained as a white solid (81.6 mg, 84%). *R_f_* = 0.08 on silica gel (ethyl acetate:petroleum ether = 1:8, v/v). mp 108–110 °C. The ee was determined to be 43% using HPLC analysis on a Chiralcel AD column (hexane/2-propanol = 90/10, 0.5 mL/min, λ = 254 nm); Retention times were 33.3 min (major) and 37.9 min (minor). [α]_D_^20^ +97.1 (*c* 1.00, CHCl_3_). IR (KBr, cm^–1^): 3425(m), 2924(s), 2833(m), 1630(w), 1599(s), 1482(s), 1259(s), 1106(m), 951(w), 796(w). ^1^H NMR (400 MHz, CDCl_3_): δ 7.18–7.14 (m, 2H), 6.81–6.75 (m, 4H), 6.59 (d, *J* = 7.6 Hz, 1H), 5.96–5.92 (m, 1H), 5.15 (s, 1H), 4.67 (d, *J* = 5.2 Hz, 1H), 3.82 (s, 3H), 3.78 (s, 3H), 2.52 (s, 3H), 2.43 (br s, 1H), 2.33 (s, 3H). ^13^C NMR (100 MHz, CDCl_3_): δ 151.0, 149.7, 149.4, 138.9, 129.0, 125.1, 124.6, 122.9, 121.7, 118.1, 114.5, 111.1, 110.9, 110.5, 64.7, 58.6, 56.1, 55.7, 32.5, 21.9. MS (ESI) *m*/*z* Calcd for C_20_H_23_NO_3_ (M^+^): 325.17; Found: 326.32 (M+H)^+^. Anal. Calcd for C_20_H_23_NO_3_: C, 73.82; H, 7.12; N, 4.30. Found: C, 73.55; H, 7.10; N, 4.40.

**(1*S*,2*S*)-2-[(4-Fluorophenyl)(methyl)amino]-5,8-dimethoxy-1,2-dihydronaphthalen-1-ol (3d)**

Following the general procedure (**III**), **3d** was obtained as a white solid (74.2 mg, 65%). *R_f_* = 0.10 on silica gel (ethyl acetate:petroleum ether = 1:6, v/v). mp 119–120 °C. The ee was determined to be 45% using HPLC analysis on a Chiralcel AD column (hexane/2-propanol = 95/5, 0.5 mL/min, λ = 254 nm); Retention times were 53.19 min (major) and 59.97 min (minor). [α]_D_^20^ +76.7 (*c* 1.00, CHCl_3_). IR (KBr, cm^–1^): 3425(m), 2936(m), 2833(m), 1595(w), 1509(s), 1482(s), 1259(s), 1103(s), 798(w). ^1^H NMR (400 MHz, CDCl_3_): δ 7.13 (d, *J* = 10 Hz, 1H), 6.97–6.89 (m, 4H), 6.81–6.74 (m, 2H), 5.94–5.90 (m, 1H), 5.13 (s, 1H), 4.56 (br s, 1H), 3.81 (br s, 3H), 3.78 (br s, 3H), 2.50 (s, 4H). ^13^C NMR (100 MHz, CDCl_3_): δ 156.9, 150.9, 149.7, 146.0, 124.9, 124.5, 123.0, 121.6, 115.6, 115.4, 111.2, 110.6, 64.6, 59.9, 56.1, 55.8, 33.1. MS (ESI) *m*/*z* Calcd for C_19_H_20_FNO_3_ (M^+^): 329.14; Found: 330.20 (M+H)^+^. Anal. Calcd for C_19_H_20_FNO_3_: C, 69.29; H, 6.12; N, 4.25. Found: C, 69.08; H, 6.43; N, 4.46.

**(1*S*,2*S*)-2-[(4-Chlorophenyl)(methyl)amino]-5,8-dimethoxy-1,2-dihydronaphthalen-1-ol (3e)**

Following the general procedure (**III**), **3e** was obtained as a white solid (88.5 mg, 74%). *R_f_* = 0.13 on silica gel (ethyl acetate:petroleum ether = 1:6, v/v). mp 99–100 °C. The ee was determined to be 54% using HPLC analysis on a Chiralcel AD column (hexane/2-propanol = 95/5, 0.5 mL/min, λ = 254 nm); Retention times were 54.6 min (major) and 58.3 min (minor). [α]_D_^20^ +88.8 (*c* 1.00, CHCl_3_). IR (KBr, cm^–1^): 3432(m), 2933(m), 2886(m), 1671(w), 1594(m), 1497(s), 1250(s), 1104(m), 808(m), 715(w). ^1^H NMR (400 MHz, CDCl_3_): δ 7.18–7.13 (m, 3H), 6.87–6.75 (m, 4H), 5.93–5.89 (m, 1H), 5.11 (s, 1H), 4.60 (t, *J* = 2.4 Hz, 1H), 3.81 (s, 3H), 3.78 (s, 3H), 2.53 (s, 4H). ^13^C NMR (100 MHz, CDCl_3_): δ 151.0, 149.8, 147.9, 129.7, 128.9, 124.7, 124.4, 123.3, 121.5, 114.9, 111.3, 110.7, 64.8, 59.1, 56.1, 55.8, 32.8. MS (ESI) *m*/*z* Calcd for C_19_H_20_ClNO_3_ (M^+^): 345.11; Found: 346.22 (M+H)^+^. Anal. Calcd for C_19_H_20_ClNO_3_: C, 65.99; H, 5.83; N, 4.05. Found: C, 65.72; H, 5.99; N, 3.93.

**(1*S*,2*S*)-2-[(4-Bromophenyl)(methyl)amino]-5,8-dimethoxy-1,2-dihydronaphthalen-1-ol (3f)**

Following the general procedure (**III**), **3f** was obtained as a white solid (94.4 mg, 70%). *R_f_* = 0.18 on silica gel (ethyl acetate:petroleum ether = 1:6, v/v). mp 126–127 °C. The ee was determined to be 89% using HPLC analysis on a Chiralcel AD column (hexane/2-propanol = 95/5, 0.5 mL/min, λ = 254 nm); Retention times were 101.9 min (major) and 105.9 min (minor). [α]_D_^20^ +152.3 (*c* 1.00, CHCl_3_). IR (KBr, cm^–1^): 3433(m), 2937(m), 2835(m), 1588(s), 1494(m), 1362(w), 1259(s), 1104(m), 1006(m), 805(m), 737(w). ^1^H NMR (400 MHz, CDCl_3_): δ 7.33–7.17 (m, 3H), 6.82–6.75 (m, 4H), 5.93–5.89 (m, 1H), 5.10 (s, 1H), 5.59 (br s, 1H), 3.81 (s, 3H), 3.78 (s, 3H), 2.52 (br s, 4H). ^13^C NMR (100 MHz, CDCl_3_): δ 151.0, 149.8, 148.3, 131.8, 124.6, 124.4, 123.3, 121.5, 115.3, 111.3, 110.7, 109.0, 64.8, 58.9, 56.1, 55.7, 32.8. MS (ESI) *m*/*z* Calcd for C_19_H_20_BrNO_3_ (M^+^): 389.06; Found: 390.12 (M+H)^+^. Anal. Calcd for C_19_H_20_BrNO_3_: C, 58.47; H, 5.17; N, 3.59. Found: C, 58.11; H, 5.12; N, 3.28.

**(1*S*,2*S*)-5,8-Dimethoxy-2-(piperidin-1-yl)-1,2-dihydronaphthalen-1-ol (3g)**

Following the general procedure (**IV**), **3g** was obtained as colorless oil (64.1 mg, 72%). *R_f_* = 0.19 on silica gel (ethyl acetate:petroleum ether = 1:1, v/v). The ee was determined to be 41% using HPLC analysis on a Chiralcel AD column (hexane/2-propanol = 90/10, 0.5 mL/min, λ = 254 nm); Retention times were 14.7 min (major) and 18.7 min (minor). [α]_D_^20^ +78.6 (*c* 1.00, CHCl_3_). IR (KBr, cm^–1^): 3384(m), 2933(s), 2835(m), 1597(w), 1482(s), 1463(m), 1401(m), 1258(s), 859(w), 799(w). ^1^H NMR (400 MHz, CDCl_3_): δ 7.05 (dd, *J* = 1.2, 1.2 Hz, 1H), 6.82 (d, *J* = 7.2 Hz, 1H), 6.53 (dd, *J* = 2.0, 2.0 Hz, 1H), 6.06 (dd, *J* = 2.4, 2.8 Hz, 1H), 4.92 (d, *J* = 11.6 Hz, 1H), 3.87 (br s, 5H), 3.51 (d, *J* = 11.2 Hz, 1H), 2.83–2.78 (m, 3H), 2.56–2.50 (m, 3H), 1.71–1.56 (m, 6H). ^13^C NMR (100 MHz, CDCl_3_): δ 150.6, 149.6, 124.7, 123.4, 123.2, 121.8, 111.0, 110.5, 64.3, 61.4, 56.1, 55.9, 49.3, 24.7, 24.0. MS (ESI) *m*/*z* Calcd for C_17_H_23_NO_3_ (M^+^): 289.17; Found: 290.07 (M+H)^+^. Anal. Calcd for C_17_H_23_NO_3_: C, 70.56; H, 8.01; N, 4.84. Found: C, 70.33; H, 8.23; N, 4.65.

**(1*S*,2*S*)-2-(3,4-Dihydro-1*H*-isoquinolin-2-yl)-5,8-dimethoxy-1,2-dihydronaphthalen-1-ol (3h)**

Following the general procedure (**IV**), **3h** was obtained as a white solid (83.0 mg, 71%). *R_f_* = 0.2 on silica gel (ethyl acetate:petroleum ether = 1:2, v/v). mp 78–80 °C. The ee was determined to be 63% using HPLC analysis on a Chiralcel AD column (hexane/2-propanol = 90/10, 0.5 mL/min, λ = 254 nm); Retention times were 101.82 min (major) and 94.9 min (minor). [α]_D_^20^ +112.8 (*c* 1.00, CHCl_3_). IR (KBr, cm^–1^): 3376(m), 2925(m), 2840(w), 1599(m), 1481(m), 1462(m), 1401(w), 1258(s), 1083(m), 954(w), 897(w). ^1^H NMR (400 MHz, CDCl_3_): δ 7.13 (m, 2H), 6.91 (d, *J* = 6.8 Hz, 2H), 6.78 (dd, *J* = 2.4, 2.4 Hz, 3H), 6.04–6.00 (m, 2H), 3.89 (br s, 1H), 3.81 (s, 6H), 3.72–3.70 (m, 2H), 3.64 (br s, 1H), 3.61 (br s, 1H), 2.63–2.59 (m, 2H), 2.15 (s, 1H). ^13^C NMR (100 MHz, CDCl_3_): δ 150.8, 149.7, 135.0, 134.2, 128.7, 126.6, 125.9, 125.4, 124.8, 124.5, 122.8, 121.9, 111.1, 110.5, 63.7, 61.6, 56.2, 55.8, 51.0, 48.2, 29.7. MS (ESI) *m*/*z* Calcd for C_21_H_23_NO_3_ (M^+^): 337.17; Found: 338.08 (M+H)^+^. Anal. Calcd for C_21_H_23_NO_3_: C, 74.75; H, 6.87; N, 4.15. Found: C, 74.85; H, 6.67; N, 4.36.

**(1*S*,2*S*)-5,8-Dimethoxy-2-(4-phenylpiperazin-1-yl)-1,2-dihydronaphthalen-1-ol (3i)**

Following the general procedure (**IV**), **3i** was obtained as a white solid (100.0 mg, 79%). *R_f_* = 0.27 on silica gel (ethyl acetate:petroleum ether = 1:1, v/v). mp 118–119 °C. The ee was determined to be 54% using HPLC analysis on a Chiralcel AD column (hexane/2-propanol = 90/10, 0.5 mL/min, λ = 254 nm); Retention times were 73.8 min (major) and 80.9 min (minor). [α]_D_^20^ +222.8 (*c* 1.00, CHCl_3_). IR (KBr, cm^–1^): 3434(m), 3010(w), 2931(m), 2831(m), 1670(m), 1598(s), 1481(s), 1450(m), 1258(s), 1004(m), 953(w), 759(w). ^1^H NMR (400 MHz, CDCl_3_): δ 7.24–7.19 (m, 2H), 7.11 (br d, *J* = 12 Hz, 1H), 6.86–6.74 (m, 5H), 5.99–5.95 (m, 1H), 5.28 (br d, *J* = 12 Hz, 2H), 3.82 (s, 3H), 3.79 (s, 3H), 3.56 (d, *J* = 5.2 Hz, 1H), 3.09 (t, *J* = 4.8, 5.2 Hz, 4H), 2.84–2.78 (m, 2H), 2.56–2.50 (m, 2H). ^13^C NMR (100 MHz, CDCl_3_): δ 151.4, 150.7, 149.7, 129.0, 124.9, 124.4, 122.7, 121.8, 119.6, 116.1, 111.1, 110.4, 63.7, 61.2, 56.1, 55.8, 49.6, 48.3. MS (ESI) *m*/*z* Calcd for C_22_H_26_N_2_O_3_ (M^+^): 366.19; Found: 367.13 (M+H)^+^. Anal. Calcd for C_22_H_26_N_2_O_3_: C, 72.11; H, 7.15; N, 7.64. Found: C, 72.40; H, 7.36; N, 7.40.

**(1*S*,2*S*)-2-[4-(3,4-Dichlorophenyl)piperazin-1-yl]-5,8-dimethoxy-1,2-dihydronaphthalen-1-ol (3j)**

Following the general procedure (**IV**), **3j** was obtained as a white solid (112.9 mg, 75%). *R_f_* = 0.35 on silica gel (ethyl acetate:petroleum ether = 1:2, v/v). mp 84–85 °C. The ee was determined to be 55% using HPLC analysis on a Chiralcel AD column (hexane/2-propanol = 90/10, 0.5 mL/min, λ = 254 nm); Retention times were 92.36 min (major) and 116.48 min (minor). [α]_D_^20^ +138.8 (*c* 1.00, CHCl_3_). IR (KBr, cm^–1^): 3420(w), 2929(m), 2833(m), 1591(m), 1552(w), 1482(m), 1450(m), 1258(s), 1238(m), 1138(w), 1003(w), 797(m). ^1^H NMR (400 MHz, CDCl_3_): δ 7.17 (dd, *J* = 3.2, 3.6 Hz, 1H), 7.08 (dd, *J* = 2.0, 2.0 Hz, 1H), 6.83 (t, *J* = 2.8 Hz, 1H), 6.74 (br s, 2H), 6.60 (dd, *J* = 3.2, 2.8 Hz, 1H), 5.94–5.91 (m, 1H), 5.24 (br s, 1H), 3.78 (dd, *J* = 2.4, 4.0 Hz, 6H), 3.52 (br s, 1H), 3.01 (br s, 5H), 2.75–2.72 (m, 2H), 2.47 (br s, 2H). ^13^C NMR (100 MHz, CDCl_3_): δ 150.7, 150.7, 149.6, 132.6, 130.3, 124.8, 124.0, 122.9, 121.8, 121.7, 117.1, 115.2, 111.1, 110.5, 63.6, 61.2, 56.1, 55.9, 48.9, 48.0. MS (ESI) *m*/*z* Calcd for C_22_H_24_Cl_2_N_2_O_3_ (M^+^): 434.12; Found: 435.23 (M+H)^+^. Anal. Calcd for C_22_H_24_Cl_2_N_2_O_3_: C, 60.70; H, 5.56; N, 6.43; Found: C, 60.49; H, 5.60; N, 6.22.

**2-{4-[(1*S*,2*S*)-1-Hydroxy-6,7-dimethoxy-1,2-dihydronaphthalen-2-yl)-piperazin-1-yl}-benzonitrile (4a)**

Following the general procedure (**IV**) starting from the 1,4-dihydro-6,7-dimethoxy-1,4-epoxynaphthalene (**1c**) (50 mg, 0.2448 mmol) and using the [Ir(COD)Cl]_2_ (4.1 mg, 0.0061 mmol), (*S*)-*p*-Tol-BINAP (8.3 mg, 0.0122 mol), NH_4_I (35.7 mg, 0.2448 mmol) and the nucleophile 1-(2-cyanophenyl)-piperazine (138 mg, 0.7334 mmol), **4a** was obtained as a white solid (74 mg, 77%) by flash chromatography (ethyl acetate:petroleum ether = 1:1, v/v). *R_f_* = 0.16 on silica gel (ethyl acetate:petroleum ether = 1:1, v/v). The ee was determined to be 37% using HPLC analysis on a Chiralcel AD column, λ = 254 nm. Flow rate = 1.0 mL/min; Retention times in 10% 2-propanol in hexanes were 73.362 min (major) and 86.016 min (minor). mp 192–193 °C. [α]_D_^25^ +65.5 (*c* 37.8 mg, CHCl_3_). IR (thin film, cm^–1^): 3512(br), 3018(w), 2927(s), 2809(s), 2271(w), 1635(w), 1490(s), 1445(s), 1351(s), 1297(s), 1135(s), 1078(s), 935(s), 846(s). ^1^H NMR (400 MHz, CDCl_3_): δ 7.57 (dd, *J* = 1.2, 1.2 Hz, 1H), 7.48 (t, *J* = 7.2 Hz, 1H), 7.13 (s, 1H), 7.03–6.99 (m, 2H), 6.45 (s, 1H), 6.47 (dd, *J* = 2.4, 2.4 Hz, 1H), 6.07 (dd, *J* = 2.4, 2.4 Hz, 1H), 4.86 (d, *J* = 11.2 Hz, 1H), 3.93 (s, 3H), 3.87 (s, 3H), 3.45 (d, *J* = 7.2 Hz, 1H), 3.28–3.22 (m, 4H), 3.15 (br, 1H), 2.99–2.98 (m, 2H), 2.78–2.77 (m, 2H). ^13^C NMR (100 MHz, CDCl_3_): δ 155.7, 148.8, 148.2, 134.5, 134.0, 129.9, 129.1, 124.8, 122.1, 118.6, 110.2, 108.8, 106.2, 68.0, 67.8, 56.2, 56.2, 52.2, 49.2; MS (ESI) *m*/*z* (M^+^): 392.10; Anal. Calcd for C_23_H_25_N_3_O_3_: C, 70.57; H, 6.44; N, 10.73. Found: C, 70.46; H, 6.67; N, 10.55.

**(1*S*,2*S*)-2-[4-(4-Fluorophenyl)piperazin-1-yl]-6,7-dimethoxy-1,2-dihydronaphthalen-1-ol (4b)**

Following the general procedure (**IV**) starting from the 1,4-dihydro-6,7-dimethoxy-1,4-epoxynaphthalene (**1c**) (50 mg, 0.2448 mmol) and using the [Ir(COD)Cl]_2_ (4.1 mg, 0.0061 mmol), (*S*)-*p*-Tol-BINAP (8.3 mg, 0.0122 mol), NH_4_I (35.7 mg, 0.2448 mmol) and the nucleophile 1-(4-fluoro-phenyl)-piperazine (132 mg, 0.7334 mmol), **4b** was obtained as a white solid (69 mg, 73%) by flash chromatography (ethyl acetate:petroleum ether = 1:1, v/v). *R_f_* = 0.20 on silica gel (ethyl acetate:petroleum ether = 1:1, v/v). The ee was determined to be 49% using HPLC analysis on a Chiralcel AD column, λ = 254 nm. Flow rate = 1.0 mL/min; Retention times in 10% 2-propanol in hexanes were 40.463 min (major) and 42.917 min (minor). mp 200–201 °C. [α]_D_^25^ +79.1 (*c* 41.2 mg, CHCl_3_). IR (thin film, cm^–1^): 3516(br), 3022(w), 2972(s), 2935(s), 2806(s), 1635(w), 1490(s), 1445(s), 1351(s), 1298(s), 1135(s), 1078(s), 935(s), 846(s). ^1^H NMR (400 MHz, CDCl_3_): δ 7.15 (s, 1H), 6.97 (d, *J* = 8.4 Hz, 2H), 6.91–6.87 (m, 2H), 6.66 (s, 1H), 6.49 (dd, *J* = 2.4, 2.4 Hz, 1H), 6.03 (dd, *J* = 2.8, 2.4 Hz, 1H), 4.87 (d, *J* = 11.2 Hz, 1H), 3.94 (s, 3H), 3.88 (s, 3H), 3.49 (dt, *J* = 11.2, 2.4 Hz, 1H), 3.25 (br, 1H), 3.18–3.13 (m, 4H), 2.96–2.94 (m, 2H), 2.74–2.73 (m, 2H). ^13^C NMR (100 MHz, CDCl_3_): δ 158.7, 156.3, 148.9, 148.3, 148.1, 130.0, 129.2, 124.8, 122.5, 118.2, 115.8, 115.6, 110.3, 108.9, 67.9, 67.8, 56.3, 56.2, 51.0, 49.2; MS (ESI) *m*/*z* (M^+^): 385.07; Anal. Calcd for C_22_H_25_FN_2_O_3_: C, 68.73; H, 6.55; N, 7.29. Found: C, 68.56; H, 6.78; N, 7.19.

**(1*S*,2*S*)-2-[4-(2-Fluorophenyl)piperazin-1-yl]-6,7-dimethoxy-1,2-dihydronaphthalen-1-ol (4c)**

Following the general procedure (**IV**) starting from the 1,4-dihydro-6,7-dimethoxy-1,4-epoxynaphthalene (**1c**) (50 mg, 0.2448 mmol) and using the [Ir(COD)Cl]_2_ (4.1 mg, 0.0061 mmol), (*S*)-BINAP (7.6 mg, 0.0122 mol), NH_4_I (35.7 mg, 0.2448 mmol) and the nucleophile 1-(2-fluoro-phenyl)-piperazine (132 mg, 0.7334 mmol), **4c** was obtained as a white solid (74 mg, 79%) by flash chromatography (ethyl acetate:petroleum ether = 1:1, v/v). *R_f_* = 0.29 on silica gel (ethyl acetate:petroleum ether = 1:1, v/v). The ee was determined to be 38% using HPLC analysis on a Chiralcel AD column, λ = 254 nm. Flow rate= 0.5 mL/min; Retention times in 10% 2-propanol in hexanes were 55.594 min (minor) and 60.269 min (major). mp 160–161 °C. [α]_D_^25^ 70.9 (*c* 35.9 mg, CHCl_3_). IR (thin film, cm^–1^): 3507(br), 3188(w), 2968(s), 2890(s), 2811(s), 1610(w), 1458(s), 1379(s), 1281(s), 1183(s), 1099(s), 935(s), 846(s). ^1^H NMR (400 MHz, CDCl_3_): δ 7.14 (s, 1H), 7.06–6.93 (m, 4H), 6.65 (s, 1H), 6.47 (dd, *J* = 2.4, 2.0 Hz, 1H), 6.05 (dd, *J* = 2.4, 2.4 Hz, 1H), 4.86 (d, *J* = 11.6 Hz, 1H), 3.93 (s, 3H), 3.87 (s, 3H), 3.46 (d, *J* = 11.2 Hz, 1H), 3.22 (br, 1H), 3.15–3.11 (m, 4H), 2.98–2.95 (m, 2H), 2.76–2.72 (m, 2H). ^13^C NMR (100 MHz, CDCl_3_): δ 148.8, 148.2, 130.0, 129.0, 124.8, 124.6, 122.7, 119.1, 119.1, 116.4, 116.2, 110.1, 108.8, 67.9, 67.8, 56.2, 56.2, 51.2, 51.2, 49.2; MS (ESI) *m*/*z* (M^+^): 385.10; Anal. Calcd for C_22_H_25_FN_2_O_3_: C, 68.73; H, 6.55; N, 7.29. Found: C, 68.62; H, 6.83; N, 7.18.

**(1*S*,2*S*)-6,7-Dimethoxy-2-[4-(4-methoxyphenyl)piperazin-1-yl]-1,2-dihydronaphthalen-1-ol (4d)**

Following the general procedure (**IV**) starting from the 1,4-dihydro-6,7-dimethoxy-1,4-epoxynaphthalene (**1c**) (50 mg, 0.2448 mmol) and using the [Ir(COD)Cl]_2_ (4.1 mg, 0.0061 mmol), (*S*)-*p*-Tol-BINAP (8.3 mg, 0.0122 mol), NH_4_I (35.7 mg, 0.2448 mmol) and the nucleophile 1-(4-methoxy-phenyl)-piperazine (132 mg, 0.7334 mmol) (141 mg, 0.7334 mmol), **4d** was obtained as a white solid (60 mg, 62%) by flash chromatography (ethyl acetate:petroleum ether = 1:1, v/v). *R_f_* = 0.20 on silica gel (ethyl acetate:petroleum ether = 1:1, v/v). The ee was determined to be 59% using HPLC analysis on a Chiralcel AD column, λ = 254 nm. Flow rate = 0.5 mL/min; Retention times in 5% 2-propanol in hexanes were 92.153 min (major) and 110.068 min (minor). mp 174–175 °C. [α]_D_^25^ +77.4 (*c* 26.5 mg, CHCl_3_). IR (thin film, cm^–1^): 3696(br), 3036(w), 2924(s), 2877(s), 1682(s), 1512(s), 1452(s), 1383(w), 1244(s), 1111(s), 1013(s), 935(w), 815(s). ^1^H NMR (400 MHz, CDCl_3_): δ 7.15 (s, 1H), 6.92 (d, *J* = 2.0 Hz, 2H), 6.85 (d, *J* = 2.0 Hz, 2H), 6.66 (s, 1H), 6.48 (dd, *J* = 2.8, 2.4 Hz, 1H), 6.05 (dd, *J* = 2.4, 2.8 Hz, 1H), 4.86 (d, *J* = 11.2 Hz, 1H), 3.94 (s, 3H), 3.88 (s, 3H), 3.77 (s, 3H), 3.48 (dt, *J* = 11.2, 2.4 Hz, 1H), 3.25 (br, 1H), 3.15–3.10 (m, 4H), 2.98–2.93 (m, 2H), 2.75–2.70 (m, 2H). ^13^C NMR (100 MHz, CDCl_3_): δ 154.2, 148.9, 148.3, 145.9, 130.1, 129.1, 124.8, 122.8, 118.6, 114.7, 110.3, 108.9, 68.0, 67.9, 56.3, 56.3, 51.5, 49.3; MS (ESI) *m*/*z* (M^+^): 397.14; Anal. Calcd for C_23_H_28_N_2_O_4_: C, 69.67; H, 7.12; N, 7.07. Found: C, 69.89; H, 7.34; N, 7.16.

**(1*S*,2*S*)-2-[4-(3,4-Dichlorophenyl)piperazin-1-yl]-6,7-dimethoxy-1,2-dihydronaphthalen-1-ol (4e)**

Following the general procedure (**IV**) starting from the 1,4-dihydro-6,7-dimethoxy-1,4-epoxynaphthalene (**1c**) (50 mg, 0.2448 mmol) and using the [Ir(COD)Cl]_2_ (4.1 mg, 0.0061 mmol), (*S*)-*p*-Tol-BINAP (8.3 mg, 0.0122 mol), NH_4_I (35.7 mg, 0.2448 mmol) and the nucleophile 1-(3,4-dichloro-phenyl)-piperazine (170 mg, 0.7334 mmol), **4e** was obtained as a white solid (50 mg, 47%) by flash chromatography (ethyl acetate:petroleum ether = 2:3, v/v). *R_f_* = 0.20 on silica gel (ethyl acetate:petroleum ether = 2:3, v/v). The ee was determined to be 16% using HPLC analysis on a Chiralcel AD column, λ = 254 nm. Flow rate = 1.0 mL/min; Retention times in 10% 2-propanol in hexanes were 44.985 min (major) and 49.376 min (minor). mp 187–188 °C. [α]_D_^25^ +25.2 (*c* 11.9 mg, CHCl_3_). IR (thin film, cm^–1^): 3591(br), 3210(w), 2973(s), 2933(s), 2874(s), 2806(s), 1676(s), 1490(s), 1445(s), 1415(s), 1383(s), 1297(m), 1077(s), 1044(s), 935(s), 846(s). ^1^H NMR (400 MHz, CDCl_3_): δ 7.28 (dd, *J* = 2.8, 2.8 Hz, 1H), 7.15 (s, 1H), 6.97 (t, *J* = 2.8 Hz, 1H), 6.75 (dt, *J* = 8.8, 2.7 Hz, 1H), 6.66 (d, *J* = 2.4 Hz, 1H), 6.50 (dd, *J* = 2.8, 2.4 Hz, 1H), 6.01 (dd, *J* = 2.8, 2.4 Hz, 1H), 4.86 (d, *J* = 11.6 Hz, 1H), 3.95 (d, *J* = 2.4 Hz, 3H), 3.89 (*J* = 2.4 Hz, 3H), 3.49 (dd, *J* = 2.4, 2.4 Hz, 1H), 3.24–3.16 (m, 5H), 2.95–2.91 (m, 2H), 2.73–2.69 (m, 2H). ^13^C NMR (100 MHz, CDCl_3_): δ 150.8, 148.9, 148.3, 133.0, 130.6, 129.9, 129.3, 124.7, 122.5, 122.2, 117.5, 115.6, 110.3, 109.0, 68.0, 67.7, 56.2, 56.2, 49.4, 48.9; MS (ESI) *m*/*z* (M^+^): 435.04; Anal. Calcd for C_22_H_24_Cl_2_N_2_O_3_: C, 60.70; H, 5.56; N, 6.43. Found: C, 60.57; H, 5.84; N, 6.36.

**Crystal structure and data of 3f**

**(1*S*,2*S*)-2-[(4-Bromophenyl)(methyl)amino]-5,8-dimethoxy-1,2-dihydronaphthalen-1-ol (3f)**


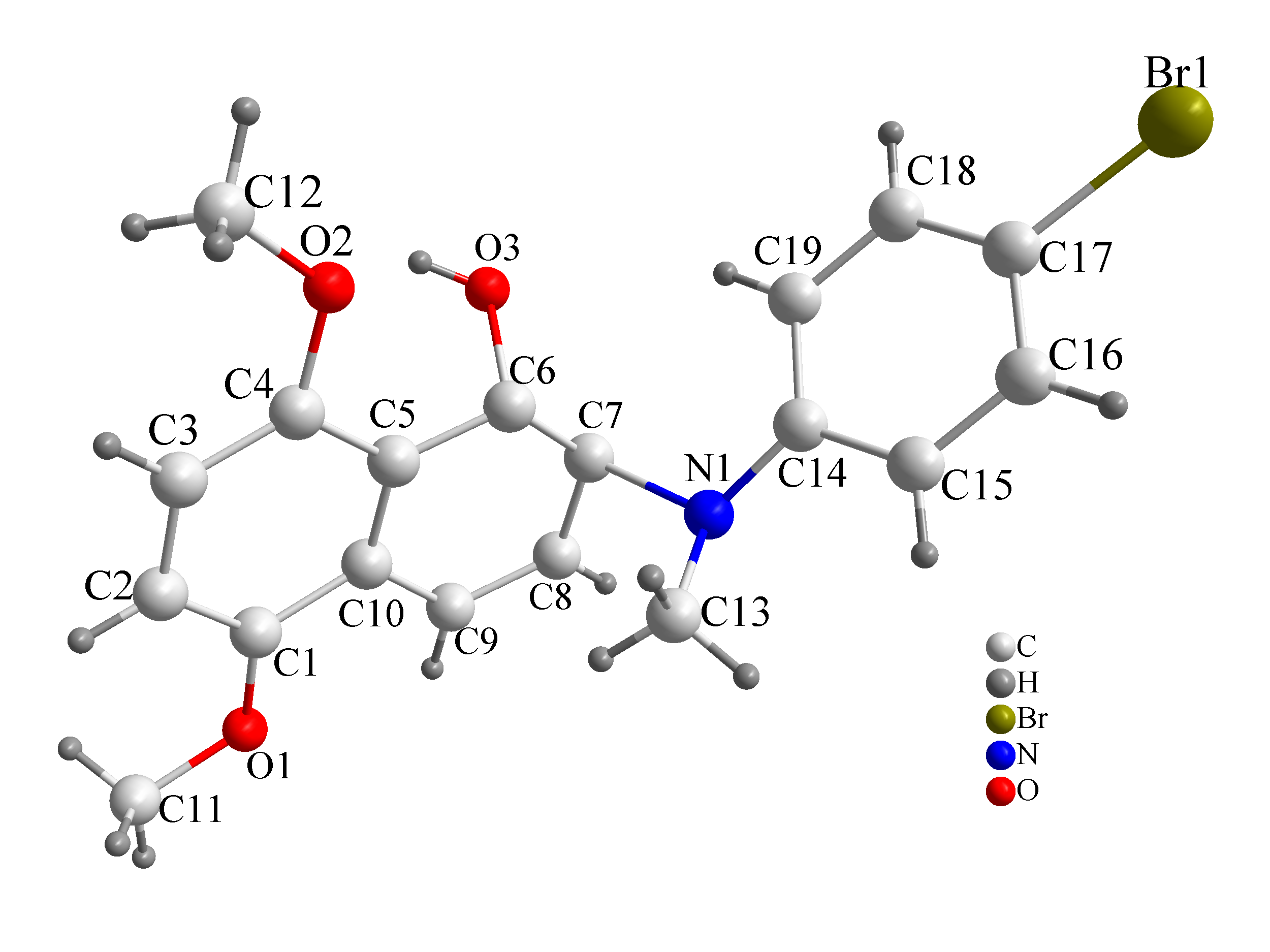


**Table 1:** Crystal data and structure refinement for **3f**.

Identification code **3f**

Empirical formula C_19_H_20_BrNO_3_

Formula weight 390.26

Temperature 293(2) K

Wavelength 0.71073 Å

Crystal system Orthorhombic

Space group P 21 21 21

Unit cell dimensions a = 10.0022(3) Å α= 90°.

b = 16.6246(6) Å β = 90°.

c = 21.0347(6) Å γ = 90°.

Volume 3497.70(19) Å^3^

Z 8

Density (calculated) 1.482 Mg/m^3^

Absorption coefficient 2.367 mm–1

F(000) 1393

Crystal size 0.30 × 0.25 × 0.18 mm^3^

Theta range for data collection 1.56 to 25.00°.

Index ranges –7<=h<=11, –15<=k<=18, –24<=l<=20

Reflections collected 18703

Independent reflections 5436 [R(int) = 0.0720]

Completeness to theta = 25.00° 90.3%

Absorption correction Multi-scan

Max. and min. transmission 0.6759 and 0.5377

Refinement method Full-matrix least-squares on F^2^

Data / restraints / parameters 5436 / 0 / 441

Goodness-of-fit on F^2^ 0.884

Final R indices [I>2sigma(I)] R1 = 0.0359, wR2 = 0.0614

R indices (all data) R1 = 0.0822, wR2 = 0.0676

Absolute structure parameter –0.003(6)

Largest diff. peak and hole 0.282 and –0.494 e.Å–3

**Table 2:** Atomic coordinates (×10^4^) and equivalent isotropic displacement parameters (Å2 × 10^3^) for **3f**. U(eq) is defined as one third of the trace of the orthogonalized Uij tensor.

_______________________________________________________________________________

x y z U(eq)

_______________________________________________________________________________

Br(1) 8760(1) 9565(1) 6256(1) 100(1)

Br(2) –3819(1) 7619(1) 3415(1) 86(1)

C(1) 6976(4) 9493(3) 5932(3) 62(2)

C(2) 5990(5) 9918(3) 6224(2) 66(1)

C(3) 4700(4) 9884(3) 5986(2) 58(1)

C(4) 4373(4) 9416(3) 5462(2) 44(1)

C(5) 5424(4) 8984(3) 5180(2) 55(1)

C(6) 6708(4) 9021(3) 5420(2) 66(1)

C(7) 2090(4) 9926(3) 5496(2) 68(1)

C(8) 2688(4) 8788(3) 4759(2) 48(1)

C(9) 1254(4) 8536(2) 4823(2) 63(1)

C(10) 299(4) 8774(3) 4432(2) 56(1)

C(11) 543(4) 9332(3) 3918(2) 47(1)

C(12) –516(4) 9706(3) 3586(2) 57(1)

C(13) –239(5) 10231(3) 3108(2) 71(2)

C(14) 1061(5) 10408(3) 2935(2) 67(1)

C(15) 2101(5) 10055(3) 3258(2) 55(1)

C(16) 1860(4) 9508(3) 3744(2) 47(1)

C(17) 2989(4) 9094(3) 4083(2) 49(1)

C(18) –2878(4) 9815(3) 3453(2) 93(2)

C(19) 3765(5) 10789(3) 2678(2) 91(2)

C(20) –2009(3) 7622(3) 3117(2) 53(1)

C(21) –1688(4) 8016(3) 2572(2) 60(1)

C(22) –377(4) 8056(3) 2372(2) 51(1)

C(23) 633(4) 7701(3) 2725(2) 43(1)

C(24) 263(4) 7284(3) 3263(2) 60(1)

C(25) –1046(4) 7251(2) 3463(2) 63(1)

C(26) 2817(4) 7058(2) 2681(2) 56(1)

C(27) 2343(3) 8195(2) 1968(2) 41(1)

C(28) 2013(3) 7690(2) 1371(2) 40(1)

C(29) 3180(4) 7295(3) 1055(2) 39(1)

C(30) 2942(4) 6687(3) 614(2) 46(1)

C(31) 3971(4) 6281(2) 333(2) 57(1)

C(32) 5283(4) 6488(3) 494(2) 59(1)

C(33) 5547(4) 7105(3) 904(2) 50(1)

C(34) 4493(4) 7519(3) 1195(2) 44(1)

C(35) 4717(4) 8176(3) 1635(2) 52(1)

C(36) 3757(4) 8480(2) 1993(2) 55(1)

C(37) 1261(4) 5990(3) 6(2) 72(1)

C(38) 7915(4) 6965(3) 805(2) 81(2)

N(1) 3068(3) 9376(2) 5248(2) 52(1)

N(2) 2005(3) 7771(2) 2566(2) 43(1)

O(1) 3340(3) 8373(2) 3734(1) 76(1)

O(2) –1777(3) 9495(2) 3790(1) 76(1)

O(3) 3446(3) 10179(2) 3120(1) 72(1)

O(4) 1356(3) 8238(2) 944(1) 61(1)

O(5) 6808(3) 7354(2) 1080(1) 70(1)

O(6) 1595(3) 6566(2) 482(1) 64(1)

_______________________________________________________________________________

**Table 3:** Bond lengths [Å] and angles [°] for **3f**.

_____________________________________________________

Br(1)-C(1) 1.914(4)

Br(2)-C(20) 1.916(4)

C(1)-C(6) 1.359(6)

C(1)-C(2) 1.360(6)

C(2)-C(3) 1.385(5)

C(2)-H(2) 0.9300

C(3)-C(4) 1.389(5)

C(3)-H(3) 0.9300

C(4)-N(1) 1.382(4)

C(4)-C(5) 1.404(5)

C(5)-C(6) 1.382(5)

C(5)-H(5) 0.9300

C(6)-H(6) 0.9300

C(7)-N(1) 1.438(4)

C(7)-H(7A) 0.9600

C(7)-H(7B) 0.9600

C(7)-H(7C) 0.9600

C(8)-N(1) 1.468(5)

C(8)-C(9) 1.500(5)

C(8)-C(17) 1.541(5)

C(8)-H(8) 0.9800

C(9)-C(10) 1.320(5)

C(9)-H(9) 0.9300

C(10)-C(11) 1.445(5)

C(10)-H(10) 0.9300

C(11)-C(16) 1.398(5)

C(11)-C(12) 1.413(5)

C(12)-C(13) 1.361(6)

C(12)-O(2) 1.378(5)

C(13)-C(14) 1.381(5)

C(13)-H(13) 0.9300

C(14)-C(15) 1.374(5)

C(14)-H(14) 0.9300

C(15)-C(16) 1.388(6)

C(15)-O(3) 1.392(4)

C(16)-C(17) 1.503(5)

C(17)-O(1) 1.448(4)

C(17)-H(17) 0.9800

C(18)-O(2) 1.414(4)

C(18)-H(18A) 0.9600

C(18)-H(18B) 0.9600

C(18)-H(18C) 0.9600

C(19)-O(3) 1.414(5)

C(19)-H(19A) 0.9600

C(19)-H(19B) 0.9600

C(19)-H(19C) 0.9600

C(20)-C(25) 1.356(5)

C(20)-C(21) 1.359(5)

C(21)-C(22) 1.378(5)

C(21)-H(21) 0.9300

C(22)-C(23) 1.385(5)

C(22)-H(22) 0.9300

C(23)-C(24) 1.378(5)

C(23)-N(2) 1.418(4)

C(24)-C(25) 1.376(5)

C(24)-H(24) 0.9300

C(25)-H(25) 0.9300

C(26)-N(2) 1.457(4)

C(26)-H(26A) 0.9600

C(26)-H(26B) 0.9600

C(26)-H(26C) 0.9600

C(27)-N(2) 1.482(5)

C(27)-C(36) 1.493(5)

C(27)-C(28) 1.545(5)

C(27)-H(27) 0.9800

C(28)-O(4) 1.439(4)

C(28)-C(29) 1.496(5)

C(28)-H(28) 0.9800

C(29)-C(30) 1.391(5)

C(29)-C(34) 1.397(5)

C(30)-C(31) 1.366(5)

C(30)-O(6) 1.390(4)

C(31)-C(32) 1.398(5)

C(31)-H(31) 0.9300

C(32)-C(33) 1.366(5)

C(32)-H(32) 0.9300

C(33)-O(5) 1.378(4)

C(33)-C(34) 1.400(5)

C(34)-C(35) 1.449(5)

C(35)-C(36) 1.322(5)

C(35)-H(35) 0.9300

C(36)-H(36) 0.9300

C(37)-O(6) 1.427(4)

C(37)-H(37A) 0.9600

C(37)-H(37B) 0.9600

C(37)-H(37C) 0.9600

C(38)-O(5) 1.407(4)

C(38)-H(38A) 0.9600

C(38)-H(38B) 0.9600

C(38)-H(38C) 0.9600

O(1)-H(1) 0.8200

O(4)-H(4) 0.8200

C(6)-C(1)-C(2) 121.0(4)

C(6)-C(1)-Br(1) 120.1(4)

C(2)-C(1)-Br(1) 118.9(4)

C(1)-C(2)-C(3) 119.5(4)

C(1)-C(2)-H(2) 120.3

C(3)-C(2)-H(2) 120.3

C(2)-C(3)-C(4) 121.9(4)

C(2)-C(3)-H(3) 119.0

C(4)-C(3)-H(3) 119.0

N(1)-C(4)-C(3) 120.5(4)

N(1)-C(4)-C(5) 123.0(4)

C(3)-C(4)-C(5) 116.4(4)

C(6)-C(5)-C(4) 121.3(4)

C(6)-C(5)-H(5) 119.3

C(4)-C(5)-H(5) 119.3

C(1)-C(6)-C(5) 119.8(4)

C(1)-C(6)-H(6) 120.1

C(5)-C(6)-H(6) 120.1

N(1)-C(7)-H(7A) 109.5

N(1)-C(7)-H(7B) 109.5

H(7A)-C(7)-H(7B) 109.5

N(1)-C(7)-H(7C) 109.5

H(7A)-C(7)-H(7C) 109.5

H(7B)-C(7)-H(7C) 109.5

N(1)-C(8)-C(9) 111.8(3)

N(1)-C(8)-C(17) 112.1(3)

C(9)-C(8)-C(17) 111.2(3)

N(1)-C(8)-H(8) 107.1

C(9)-C(8)-H(8) 107.1

C(17)-C(8)-H(8) 107.1

C(10)-C(9)-C(8) 123.5(4)

C(10)-C(9)-H(9) 118.3

C(8)-C(9)-H(9) 118.3

C(9)-C(10)-C(11) 122.4(4)

C(9)-C(10)-H(10) 118.8

C(11)-C(10)-H(10) 118.8

C(16)-C(11)-C(12) 119.0(4)

C(16)-C(11)-C(10) 119.3(4)

C(12)-C(11)-C(10) 121.7(4)

C(13)-C(12)-O(2) 125.4(4)

C(13)-C(12)-C(11) 119.7(4)

O(2)-C(12)-C(11) 114.9(5)

C(12)-C(13)-C(14) 121.5(4)

C(12)-C(13)-H(13) 119.3

C(14)-C(13)-H(13) 119.3

C(15)-C(14)-C(13) 119.5(4)

C(15)-C(14)-H(14) 120.3

C(13)-C(14)-H(14) 120.3

C(14)-C(15)-C(16) 120.8(4)

C(14)-C(15)-O(3) 124.4(5)

C(16)-C(15)-O(3) 114.7(4)

C(15)-C(16)-C(11) 119.6(4)

C(15)-C(16)-C(17) 121.2(4)

C(11)-C(16)-C(17) 119.2(4)

O(1)-C(17)-C(16) 108.7(3)

O(1)-C(17)-C(8) 104.0(4)

C(16)-C(17)-C(8) 116.2(3)

O(1)-C(17)-H(17) 109.2

C(16)-C(17)-H(17) 109.2

C(8)-C(17)-H(17) 109.2

O(2)-C(18)-H(18A) 109.5

O(2)-C(18)-H(18B) 109.5

H(18A)-C(18)-H(18B) 109.5

O(2)-C(18)-H(18C) 109.5

H(18A)-C(18)-H(18C) 109.5

H(18B)-C(18)-H(18C) 109.5

O(3)-C(19)-H(19A) 109.5

O(3)-C(19)-H(19B) 109.5

H(19A)-C(19)-H(19B) 109.5

O(3)-C(19)-H(19C) 109.5

H(19A)-C(19)-H(19C) 109.5

H(19B)-C(19)-H(19C) 109.5

C(25)-C(20)-C(21) 120.3(3)

C(25)-C(20)-Br(2) 119.7(4)

C(21)-C(20)-Br(2) 120.0(3)

C(20)-C(21)-C(22) 120.4(4)

C(20)-C(21)-H(21) 119.8

C(22)-C(21)-H(21) 119.8

C(21)-C(22)-C(23) 120.7(4)

C(21)-C(22)-H(22) 119.7

C(23)-C(22)-H(22) 119.7

C(24)-C(23)-C(22) 117.3(3)

C(24)-C(23)-N(2) 119.7(4)

C(22)-C(23)-N(2) 123.0(4)

C(25)-C(24)-C(23) 121.8(4)

C(25)-C(24)-H(24) 119.1

C(23)-C(24)-H(24) 119.1

C(20)-C(25)-C(24) 119.6(4)

C(20)-C(25)-H(25) 120.2

C(24)-C(25)-H(25) 120.2

N(2)-C(26)-H(26A) 109.5

N(2)-C(26)-H(26B) 109.5

H(26A)-C(26)-H(26B) 109.5

N(2)-C(26)-H(26C) 109.5

H(26A)-C(26)-H(26C) 109.5

H(26B)-C(26)-H(26C) 109.5

N(2)-C(27)-C(36) 109.7(3)

N(2)-C(27)-C(28) 112.4(3)

C(36)-C(27)-C(28) 113.9(3)

N(2)-C(27)-H(27) 106.8

C(36)-C(27)-H(27) 106.8

C(28)-C(27)-H(27) 106.8

O(4)-C(28)-C(29) 110.8(3)

O(4)-C(28)-C(27) 105.1(3)

C(29)-C(28)-C(27) 115.7(3)

O(4)-C(28)-H(28) 108.3

C(29)-C(28)-H(28) 108.3

C(27)-C(28)-H(28) 108.3

C(30)-C(29)-C(34) 119.7(4)

C(30)-C(29)-C(28) 118.8(4)

C(34)-C(29)-C(28) 121.5(4)

C(31)-C(30)-O(6) 124.9(4)

C(31)-C(30)-C(29) 121.2(4)

O(6)-C(30)-C(29) 113.8(4)

C(30)-C(31)-C(32) 118.8(4)

C(30)-C(31)-H(31) 120.6

C(32)-C(31)-H(31) 120.6

C(33)-C(32)-C(31) 121.2(4)

C(33)-C(32)-H(32) 119.4

C(31)-C(32)-H(32) 119.4

C(32)-C(33)-O(5) 124.9(4)

C(32)-C(33)-C(34) 120.0(4)

O(5)-C(33)-C(34) 115.1(4)

C(29)-C(34)-C(33) 119.0(4)

C(29)-C(34)-C(35) 118.8(4)

C(33)-C(34)-C(35) 122.2(4)

C(36)-C(35)-C(34) 122.7(4)

C(36)-C(35)-H(35) 118.6

C(34)-C(35)-H(35) 118.6

C(35)-C(36)-C(27) 123.1(4)

C(35)-C(36)-H(36) 118.5

C(27)-C(36)-H(36) 118.5

O(6)-C(37)-H(37A) 109.5

O(6)-C(37)-H(37B) 109.5

H(37A)-C(37)-H(37B) 109.5

O(6)-C(37)-H(37C) 109.5

H(37A)-C(37)-H(37C) 109.5

H(37B)-C(37)-H(37C) 109.5

O(5)-C(38)-H(38A) 109.5

O(5)-C(38)-H(38B) 109.5

H(38A)-C(38)-H(38B) 109.5

O(5)-C(38)-H(38C) 109.5

H(38A)-C(38)-H(38C) 109.5

H(38B)-C(38)-H(38C) 109.5

C(4)-N(1)-C(7) 119.6(4)

C(4)-N(1)-C(8) 120.3(3)

C(7)-N(1)-C(8) 120.1(3)

C(23)-N(2)-C(26) 115.7(3)

C(23)-N(2)-C(27) 117.4(3)

C(26)-N(2)-C(27) 113.7(3)

C(17)-O(1)-H(1) 109.5

C(12)-O(2)-C(18) 117.5(4)

C(15)-O(3)-C(19) 117.5(4)

C(28)-O(4)-H(4) 109.5

C(33)-O(5)-C(38) 118.2(3)

C(30)-O(6)-C(37) 117.7(3)

_____________________________________________________________

**Table 4:** Anisotropic displacement parameters (Å2 × 10^3^) for **1**. The anisotropic displacement factor exponent takes the form: –2p^2^[ h^2^ a*^2^U^11^ + ... + 2 h k a* b* U^12^ ]

______________________________________________________________________________

U^11^ U^22^ U^33^ U^23^ U^13^ U^12^

______________________________________________________________________________

Br(1) 55(1) 131(1) 113(1) 66(1) –24(1) –23(1)

Br(2) 53(1) 133(1) 71(1) –3(1) 10(1) –8(1)

C(1) 43(3) 77(4) 66(4) 39(3) –12(3) –16(3)

C(2) 68(3) 65(3) 65(3) 9(3) –17(3) –9(3)

C(3) 58(3) 57(4) 57(4) –2(3) 1(3) 2(2)

C(4) 44(2) 49(3) 38(3) 0(3) 2(2) –2(2)

C(5) 62(3) 62(4) 40(3) 1(3) 5(2) 4(3)

C(6) 48(3) 89(4) 59(4) 16(3) 9(3) 6(3)

C(7) 54(3) 71(4) 79(4) –19(3) –6(3) 13(3)

C(8) 57(3) 54(4) 34(3) –1(3) 3(2) –3(2)

C(9) 68(3) 69(3) 51(3) 8(3) 5(3) –21(3)

C(10) 48(3) 67(4) 54(4) –13(3) 5(3) –10(2)

C(11) 57(3) 53(4) 30(3) –6(3) 2(2) 5(2)

C(12) 59(3) 61(4) 49(4) –14(3) 3(3) 6(3)

C(13) 70(3) 83(5) 58(4) –3(3) –11(3) 15(3)

C(14) 91(4) 67(4) 43(3) 10(3) 3(3) 4(4)

C(15) 72(3) 58(4) 36(4) –3(3) 7(3) –6(3)

C(16) 50(3) 55(3) 36(3) –9(3) 5(2) 1(2)

C(17) 47(2) 57(3) 42(3) –18(3) 3(2) 2(2)

C(18) 54(3) 122(5) 103(4) –29(4) –9(3) 26(3)

C(19) 113(4) 93(4) 67(4) 11(3) 19(4) –13(4)

C(20) 40(2) 66(4) 52(3) –7(3) 6(2) 2(2)

C(21) 48(3) 89(4) 44(3) 12(3) –3(2) 15(2)

C(22) 53(3) 68(4) 31(3) 13(2) 1(2) 9(2)

C(23) 46(2) 51(3) 32(3) –2(3) –4(2) 3(2)

C(24) 62(3) 70(4) 49(3) 18(3) –5(2) 18(2)

C(25) 64(3) 70(3) 55(3) 13(3) 14(3) 2(3)

C(26) 55(2) 62(4) 52(3) 6(3) –1(2) 13(2)

C(27) 51(2) 39(3) 34(3) –1(3) –1(2) 8(2)

C(28) 47(2) 41(3) 32(3) 2(3) –5(2) 9(2)

C(29) 53(2) 36(3) 28(3) 0(2) 6(2) 5(2)

C(30) 58(3) 39(3) 40(3) –9(3) 4(2) –3(2)

C(31) 76(3) 57(3) 38(3) –13(2) 10(3) 3(3)

C(32) 63(3) 66(4) 47(3) 0(3) 12(3) 17(3)

C(33) 56(3) 59(4) 34(3) 4(3) 9(2) 0(3)

C(34) 51(2) 46(3) 36(3) –3(3) 4(2) –2(2)

C(35) 53(3) 63(3) 41(3) 0(3) –1(2) –5(2)

C(36) 62(3) 56(3) 45(3) –13(2) –1(3) –12(3)

C(37) 84(3) 74(3) 59(3) –27(3) –2(3) –14(3)

C(38) 54(3) 109(4) 80(4) 20(3) 16(3) 12(3)

N(1) 55(2) 58(3) 43(2) –12(2) –6(2) 8(2)

N(2) 48(2) 44(3) 35(2) 1(2) 0(2) 9(2)

O(1) 71(2) 105(3) 53(2) –19(2) –13(2) 38(2)

O(2) 47(2) 103(3) 77(2) –6(2) –5(2) 6(2)

O(3) 73(2) 83(2) 60(2) 21(2) 15(2) –5(2)

O(4) 69(2) 73(2) 41(2) –4(2) –8(2) 9(2)

O(5) 40(2) 100(3) 70(2) –8(2) 10(2) 3(2)

O(6) 65(2) 69(2) 56(2) –27(2) 4(2) –6(2)

______________________________________________________________________________

**Table 5:** Hydrogen coordinates (×10^4^) and isotropic displacement parameters (Å2 × 10^3^) for **3f.**

_______________________________________________________________________________

x y z U(eq)

_______________________________________________________________________________

H(2) 6179 10228 6580 79

H(3) 4033 10184 6183 69

H(5) 5252 8668 4825 66

H(6) 7388 8723 5232 79

H(7A) 1915 9800 5933 81

H(7B) 1279 9881 5255 81

H(7C) 2425 10466 5465 81

H(8) 3236 8308 4827 58

H(9) 1025 8194 5156 75

H(10) –561 8576 4490 67

H(13) –940 10477 2892 85

H(14) 1231 10762 2603 81

H(17) 3764 9454 4097 59

H(18A) –2878 10391 3491 111

H(18B) –3694 9604 3627 111

H(18C) –2812 9668 3013 111

H(19A) 3395 10655 2271 109

H(19B) 4719 10836 2643 109

H(19C) 3398 11292 2820 109

H(21) –2356 8260 2333 72

H(22) –169 8324 1997 61

H(24) 916 7019 3498 72

H(25) –1268 6975 3833 76

H(26A) 2843 6948 3129 67

H(26B) 3708 7149 2528 67

H(26C) 2433 6607 2462 67

H(27) 1778 8676 1950 50

H(28) 1372 7271 1493 48

H(31) 3802 5873 41 68

H(32) 5990 6200 318 70

H(35) 5573 8391 1667 63

H(36) 3973 8892 2275 65

H(37A) 1666 6142 –390 87

H(37B) 307 5969 –44 87

H(37C) 1584 5470 131 87

H(38A) 7888 6402 906 97

H(38B) 8725 7196 968 97

H(38C) 7888 7032 352 97

H(1) 2955 8373 3390 92

H(4) 1159 8002 615 73

_______________________________________________________________________________**Table 6:** Hydrogen bonds for **3f** [Å and °].

____________________________________________________________________________

D-H...A d(D-H) d(H...A) d(D...A) <(DHA)

____________________________________________________________________________

O(1)-H(1)...N(2) 0.82 2.22 2.971(4) 153.1

O(4)-H(4)...O(6) 0.82 2.44 2.954(4) 121.5

____________________________________________________________________________

**Copies of ^1^H and ^13^C NMR spectra of 2a-l, 3a-j and 4a-d**


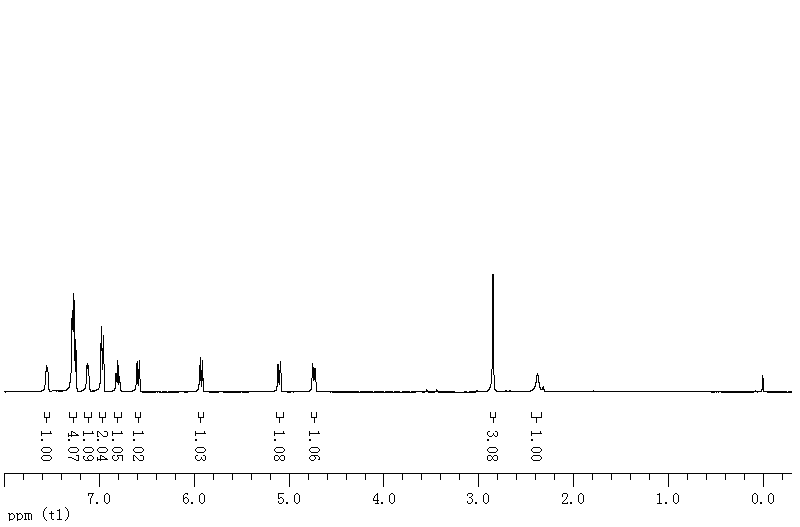


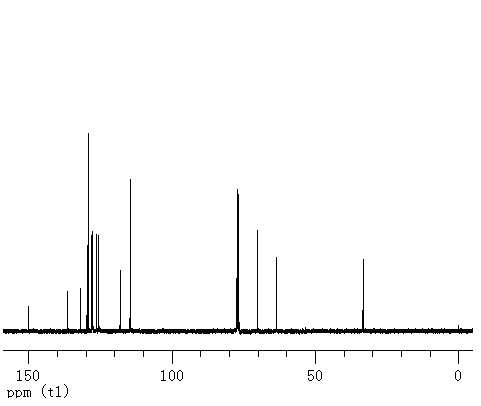


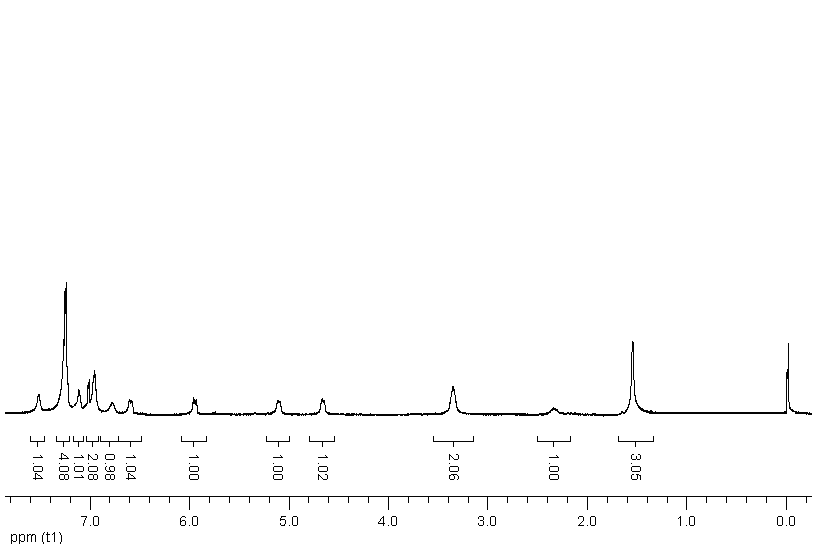


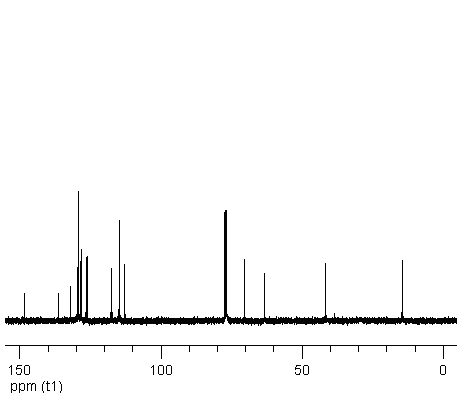


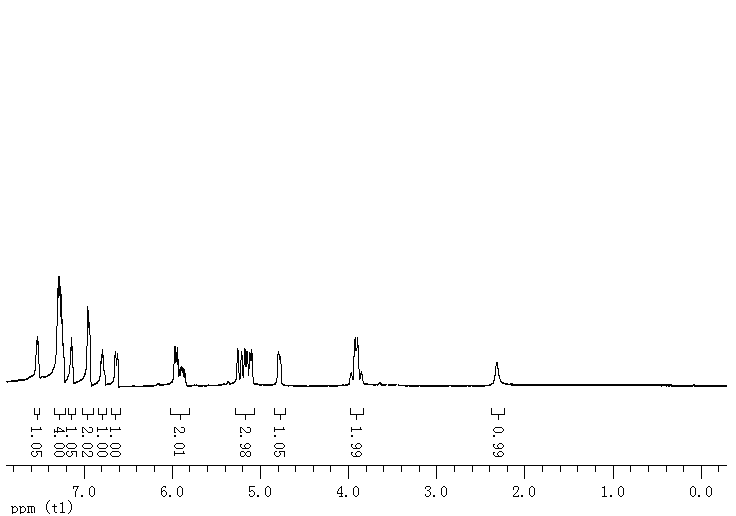


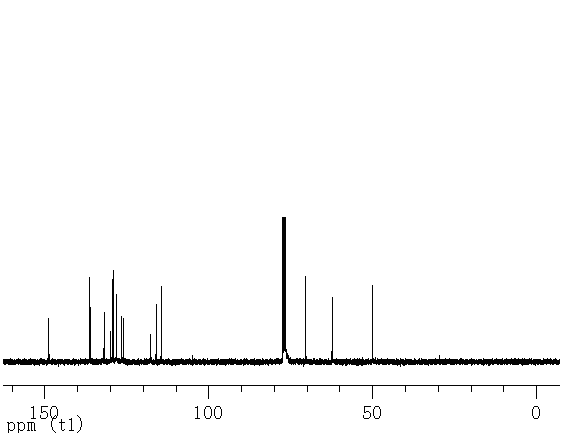


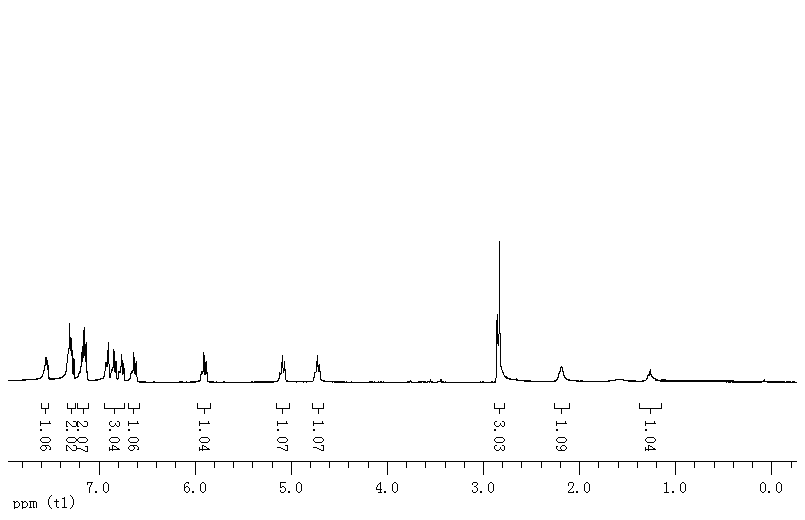


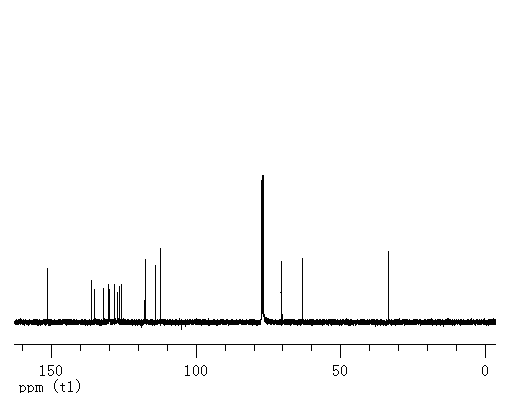


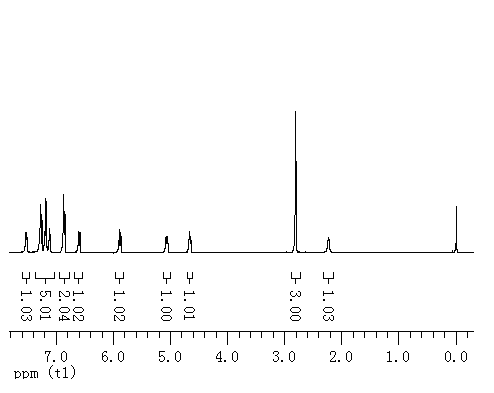


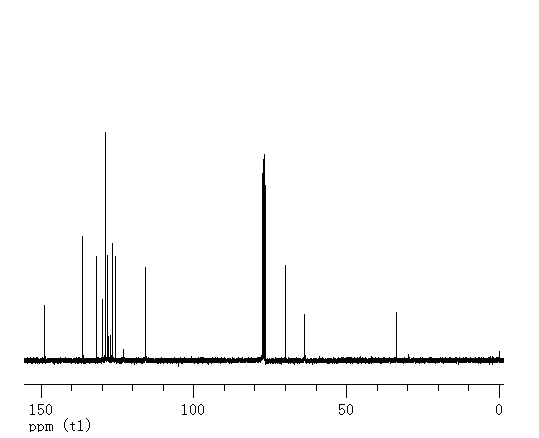


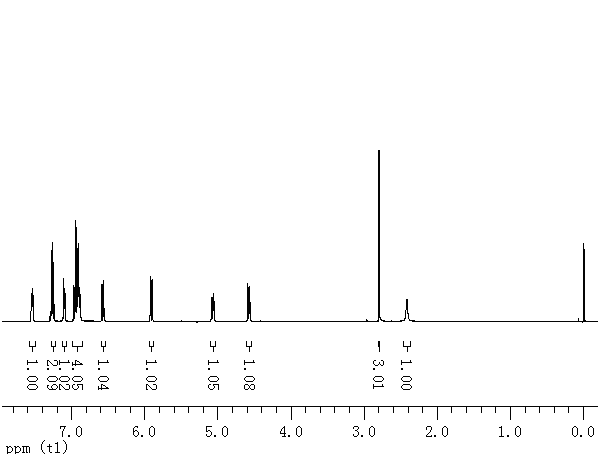


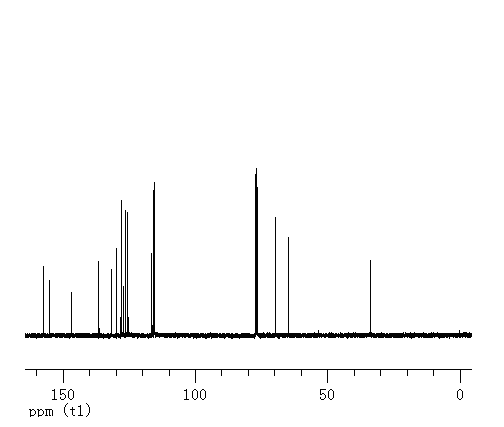


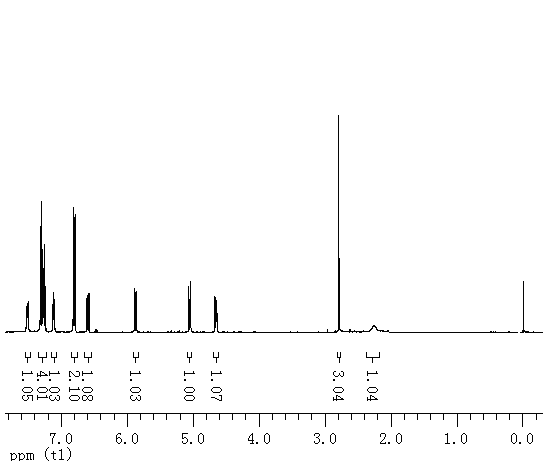


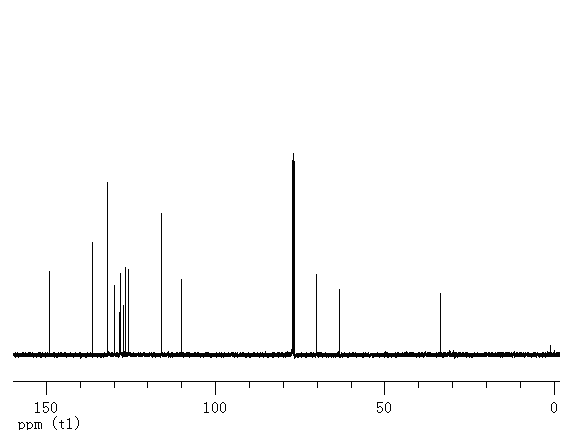


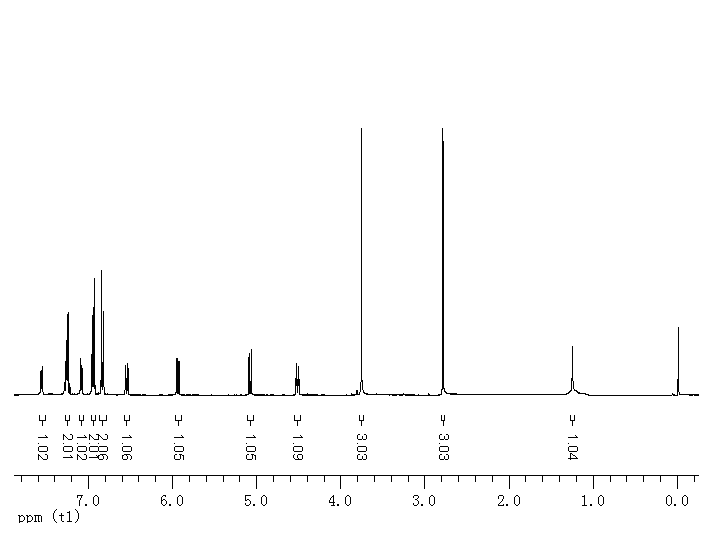


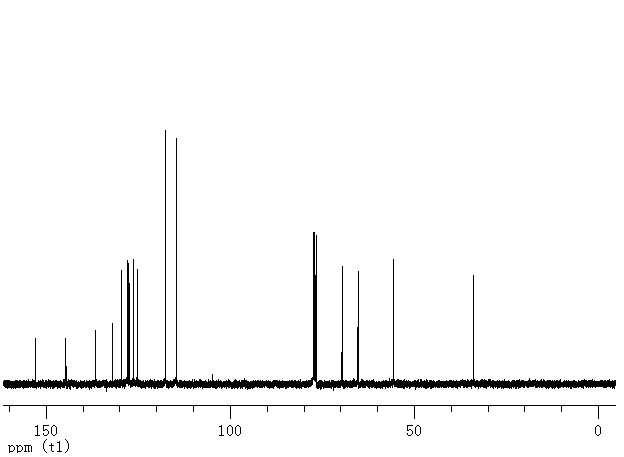


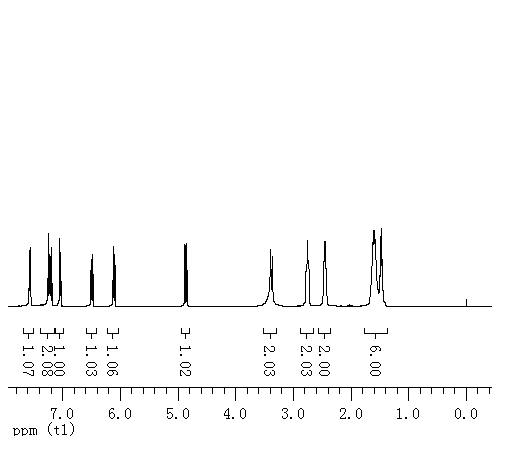


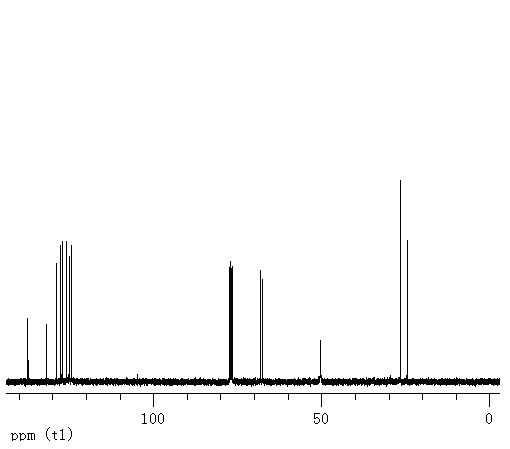


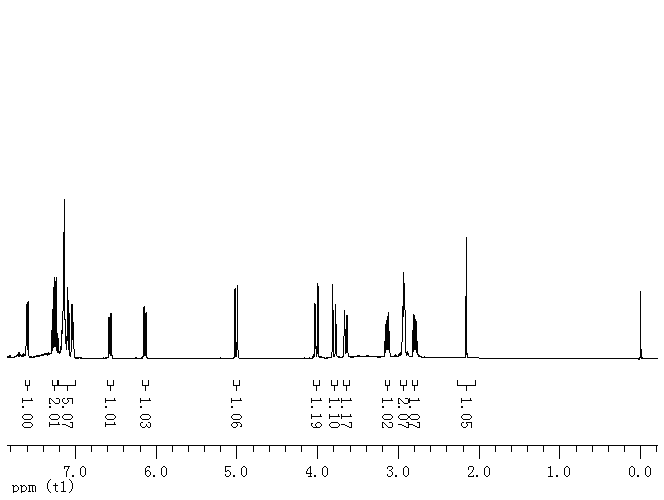


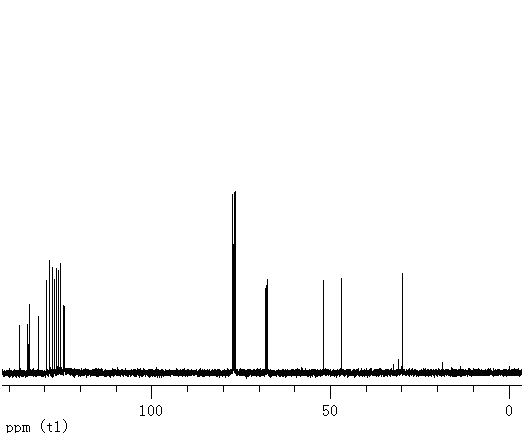


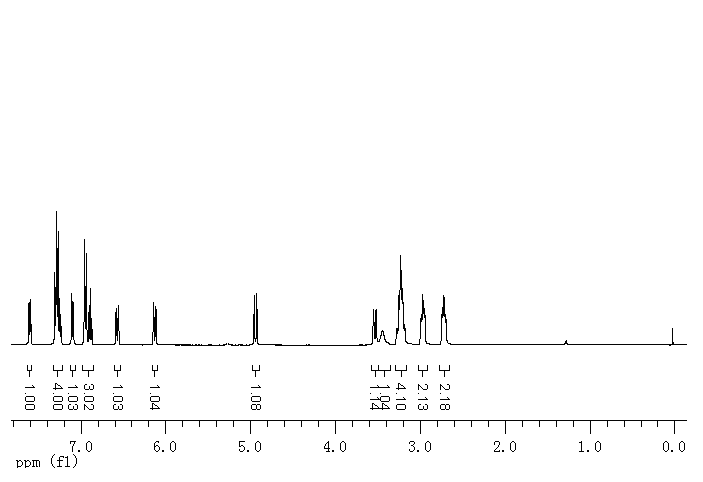


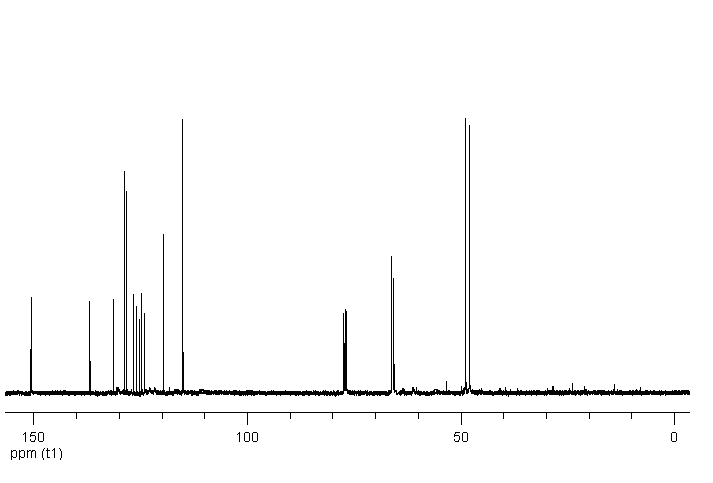


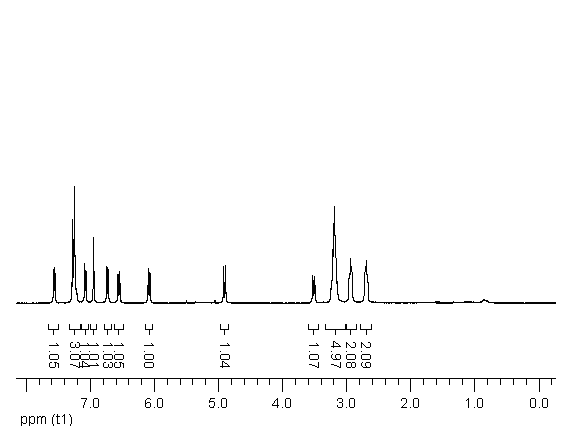


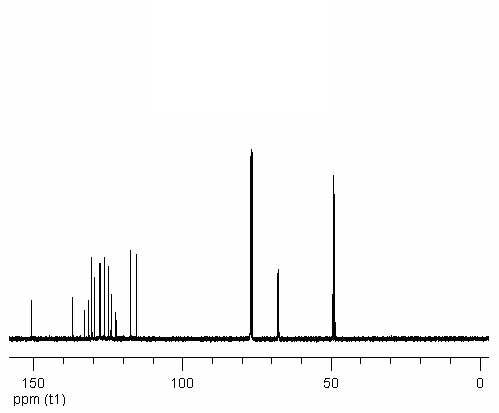


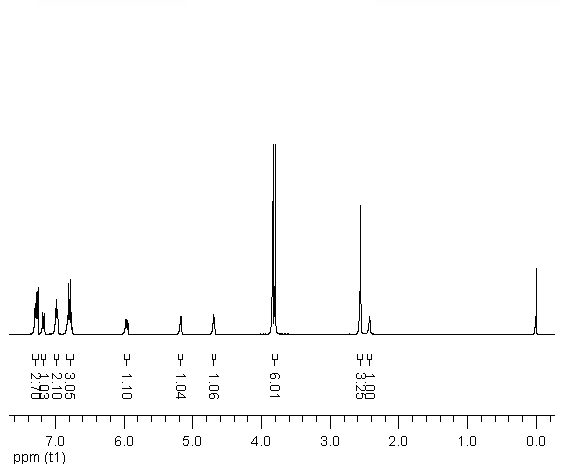


**
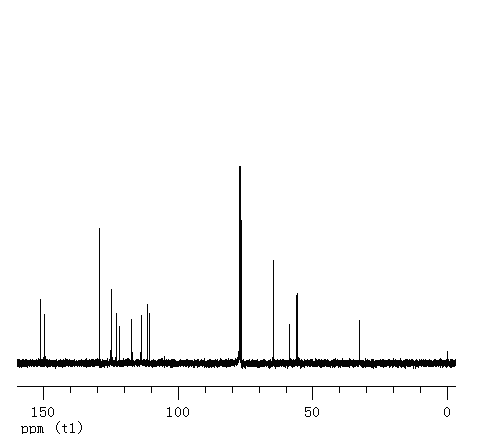
**


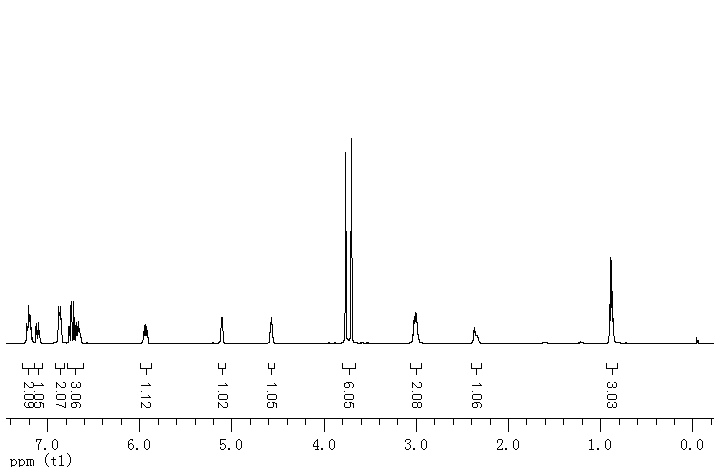


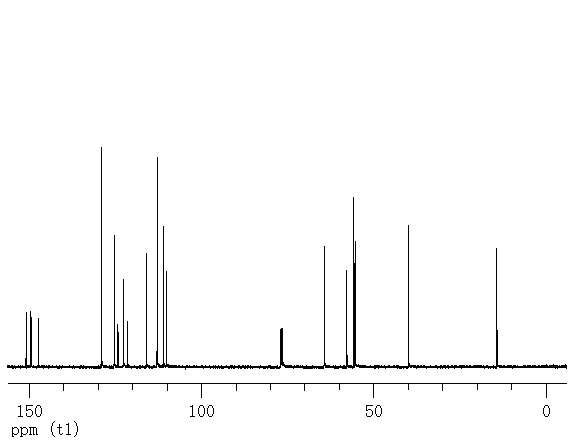


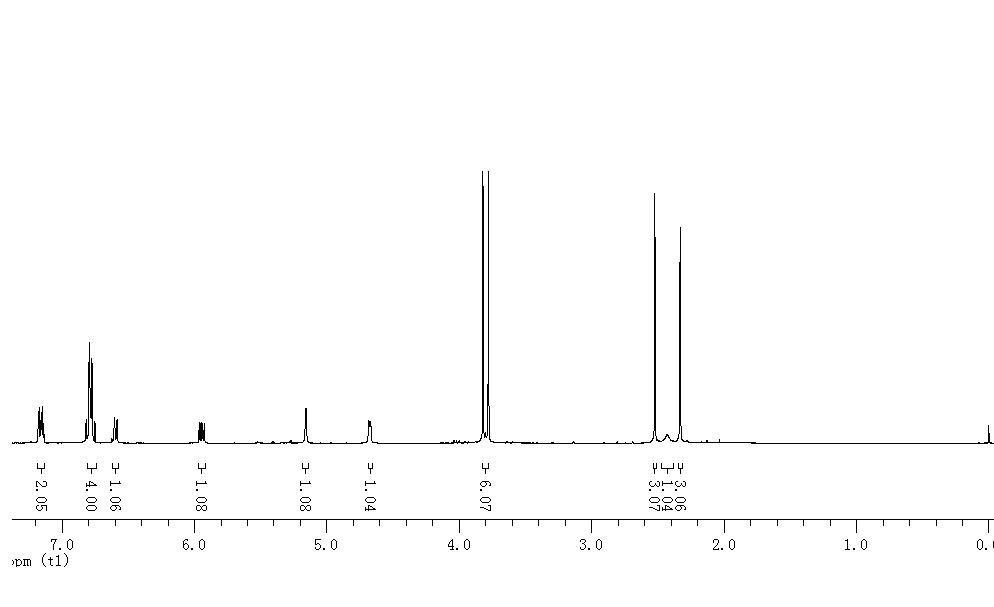


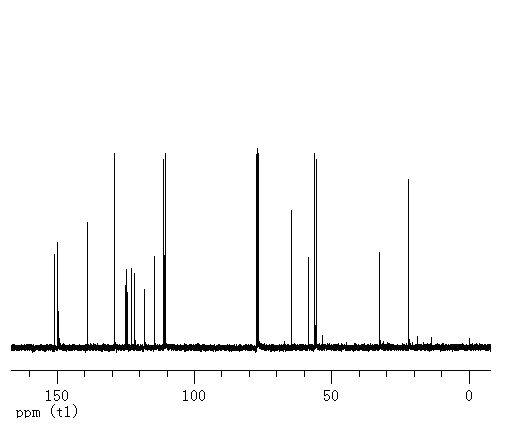


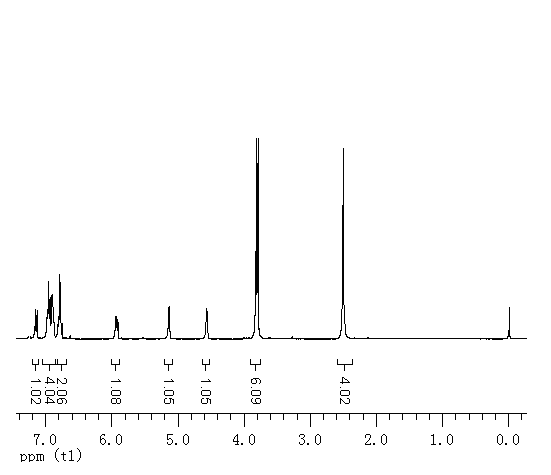


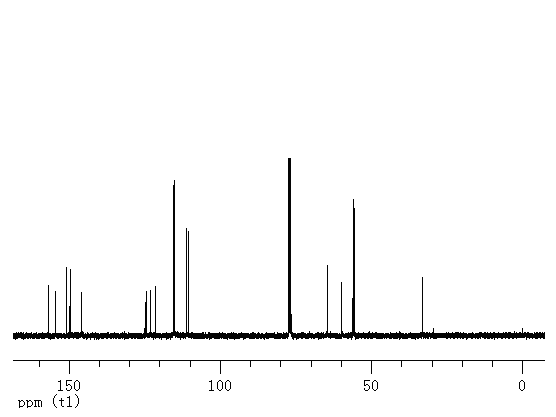


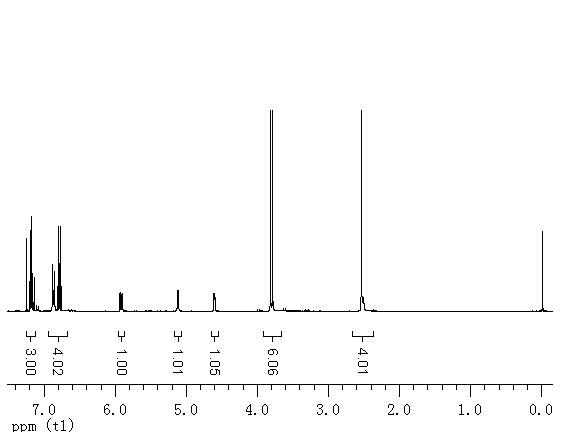


**
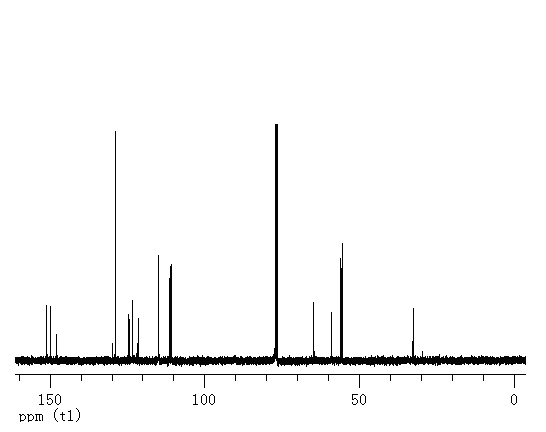
**


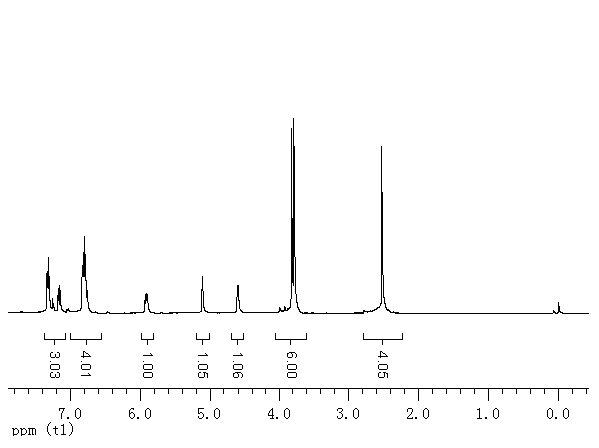


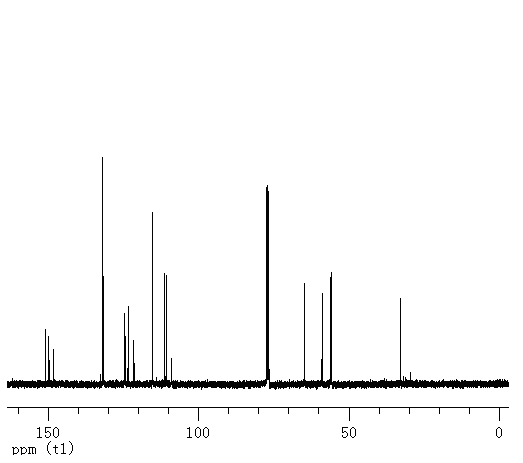


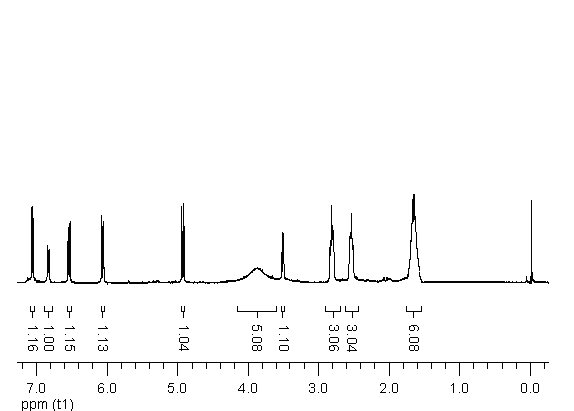


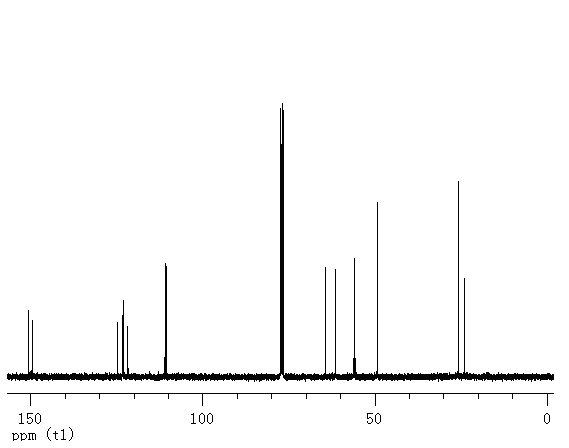


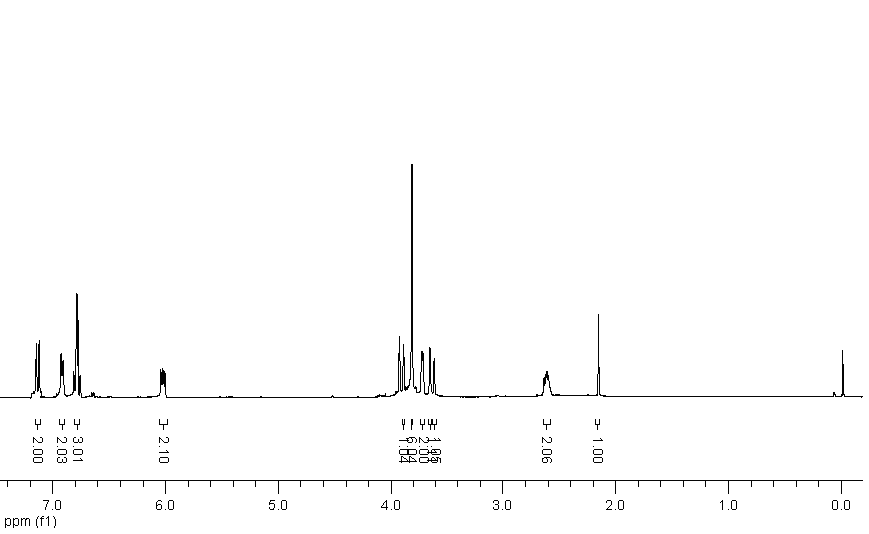


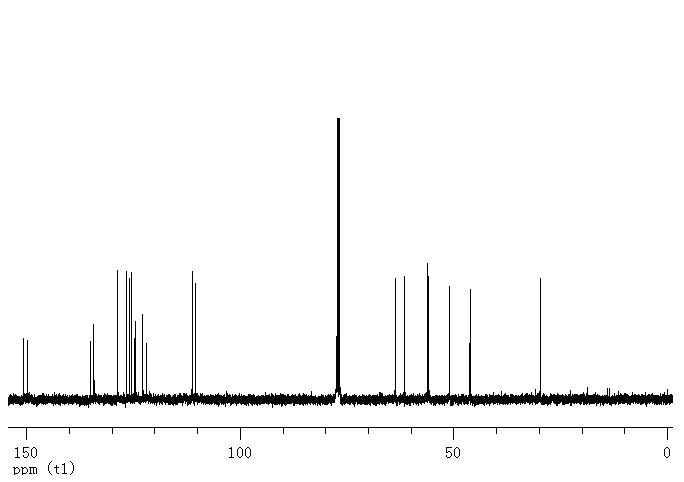


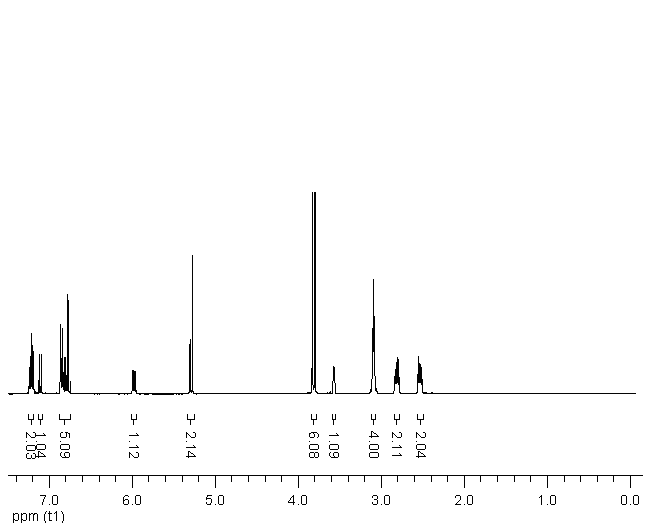


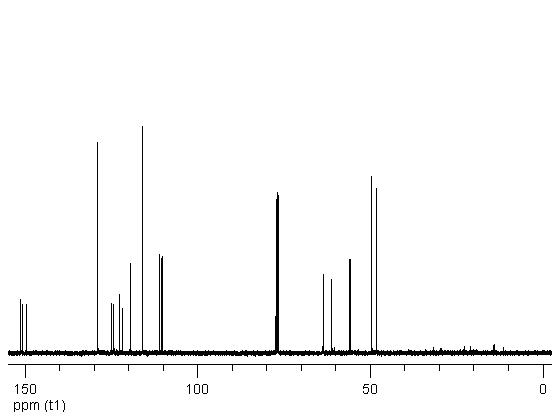


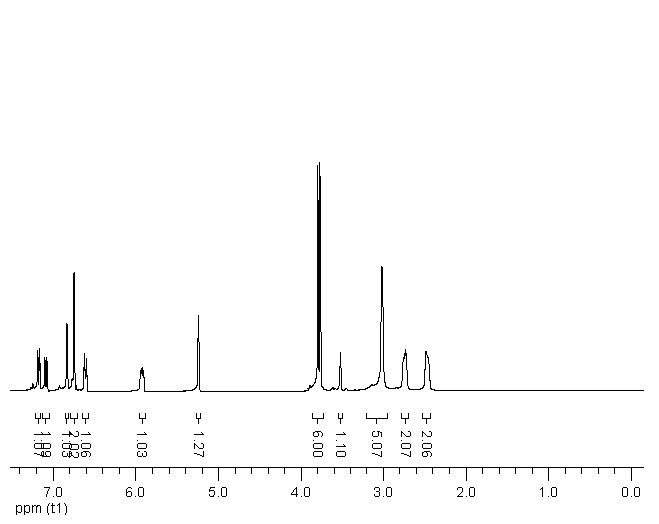


**
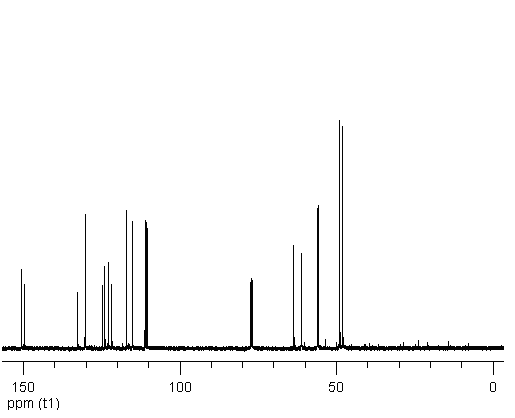
**


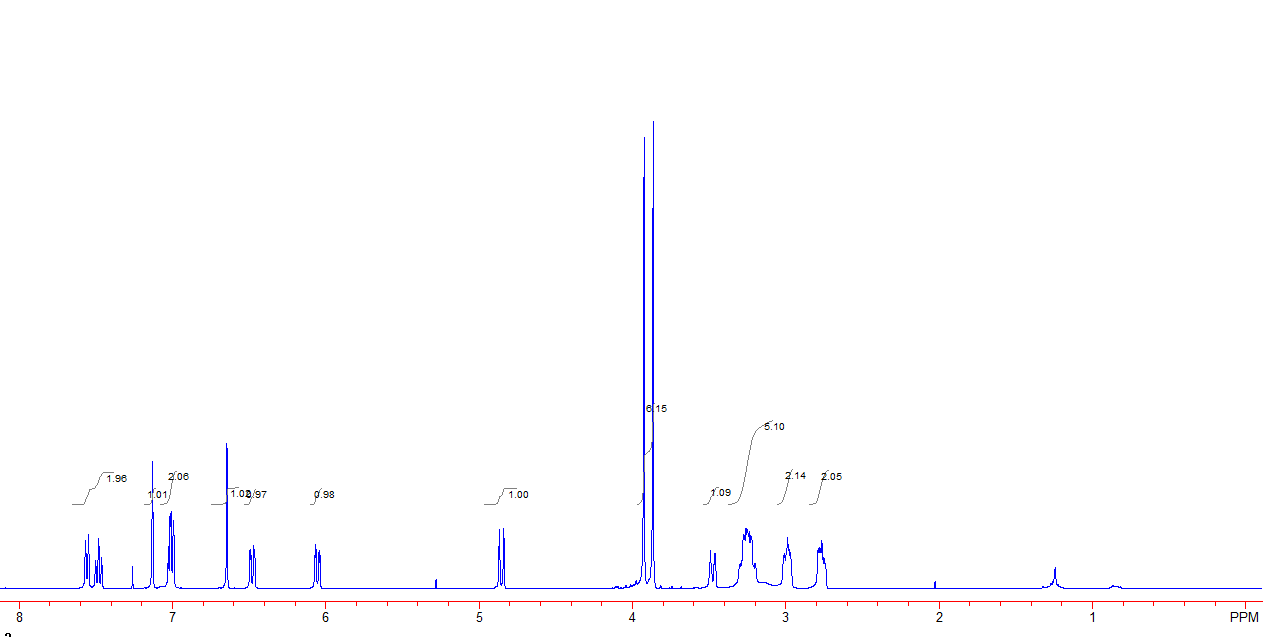


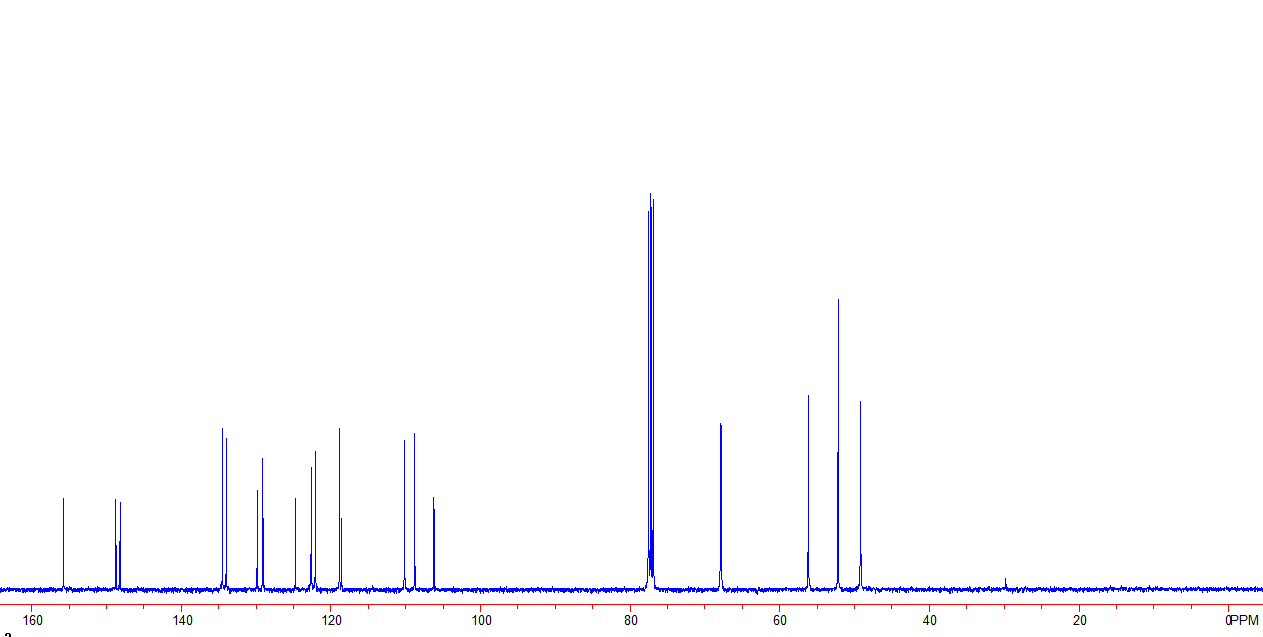


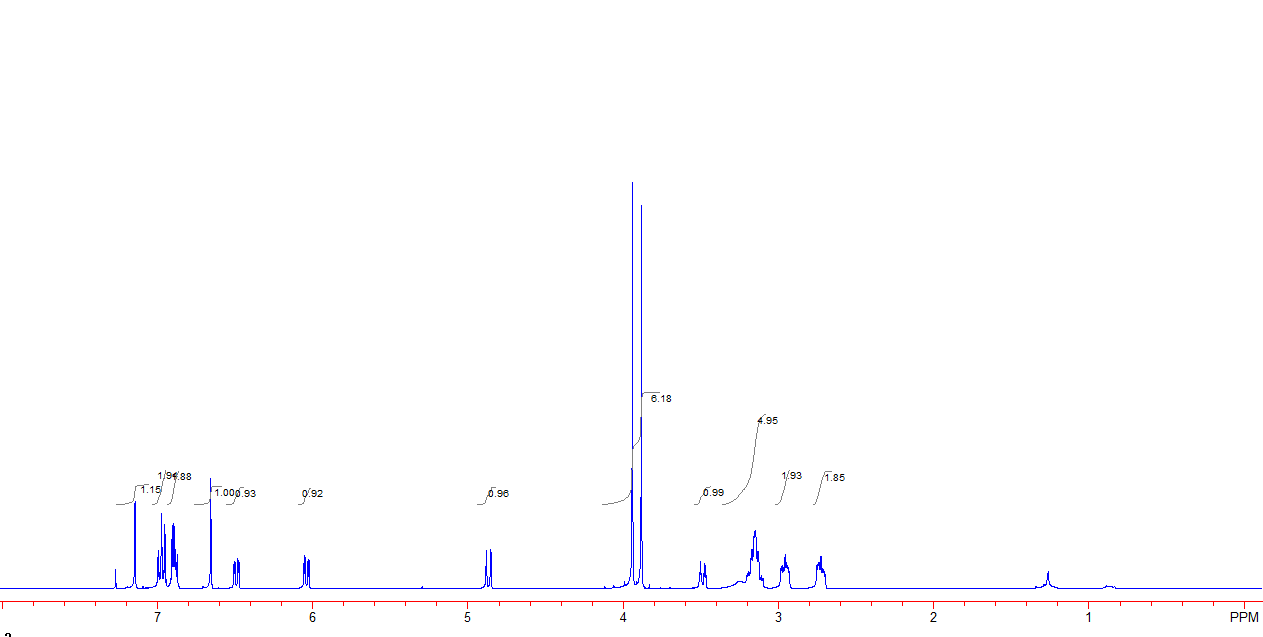


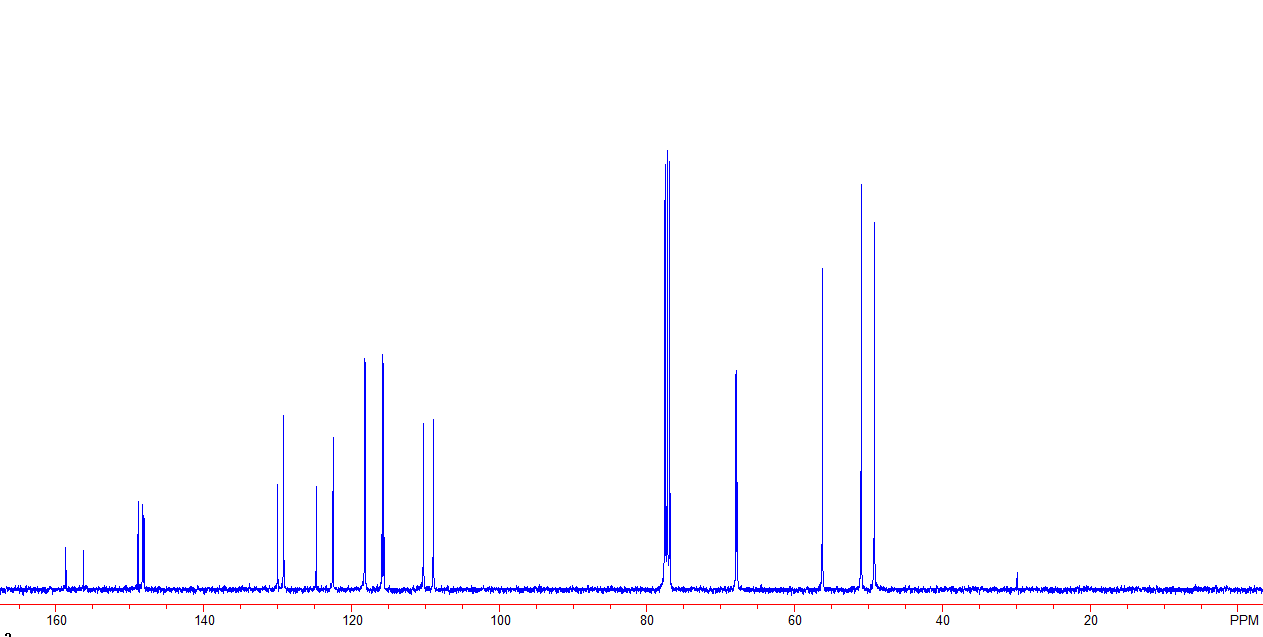


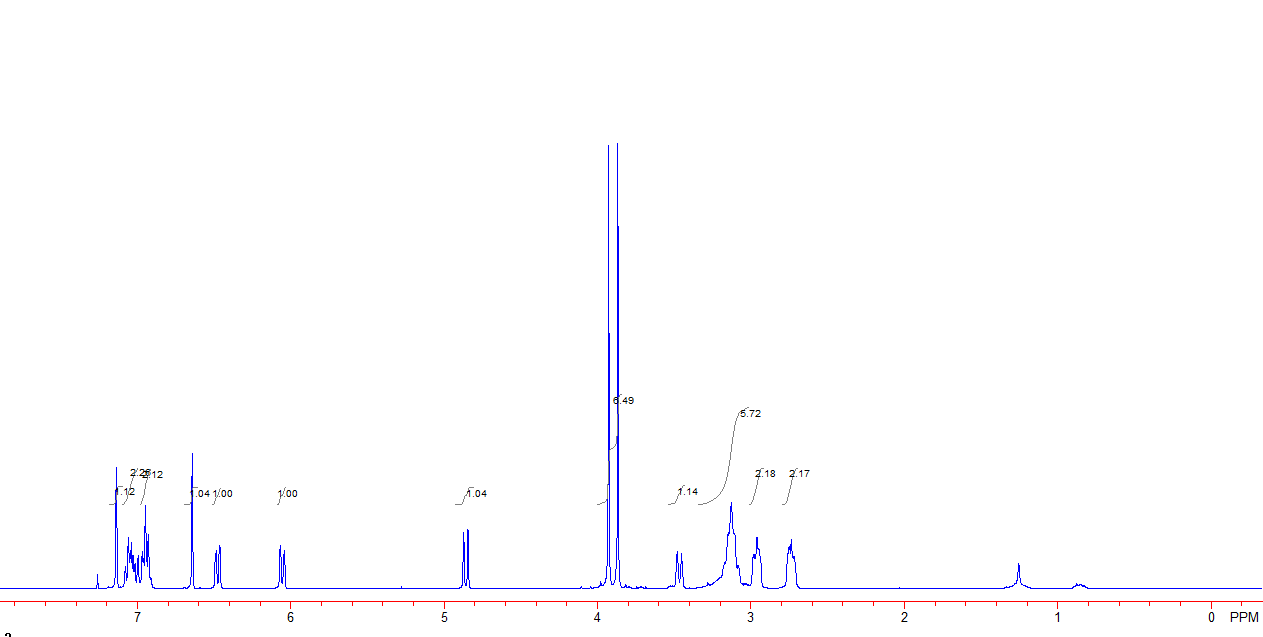


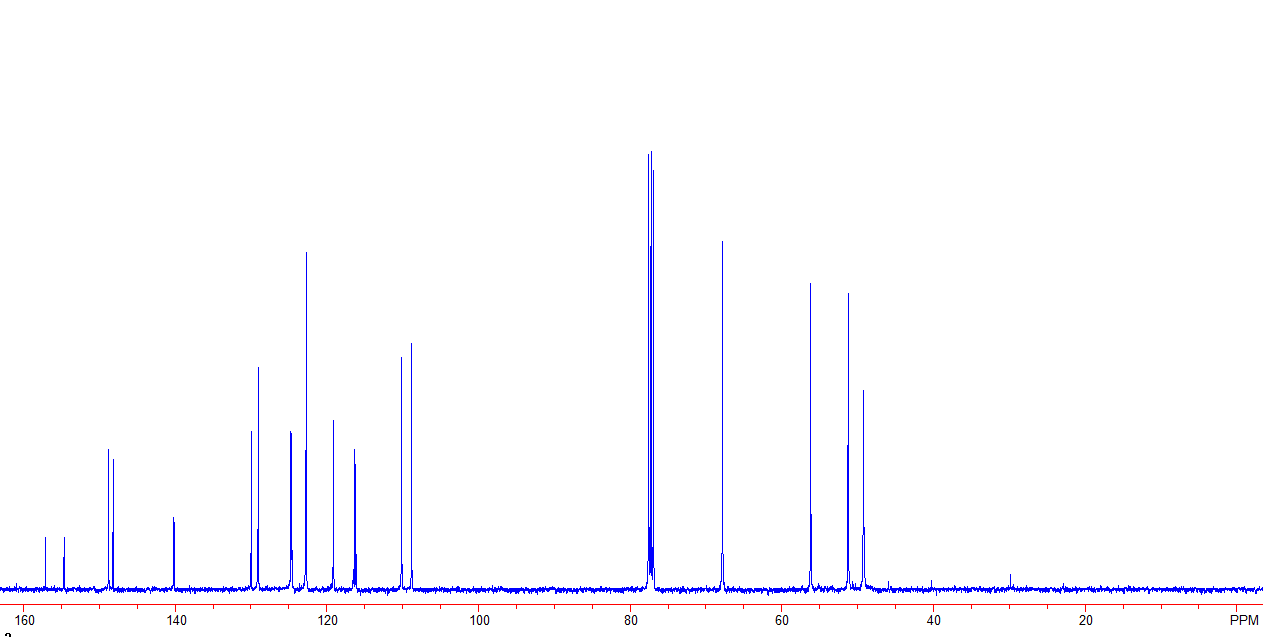


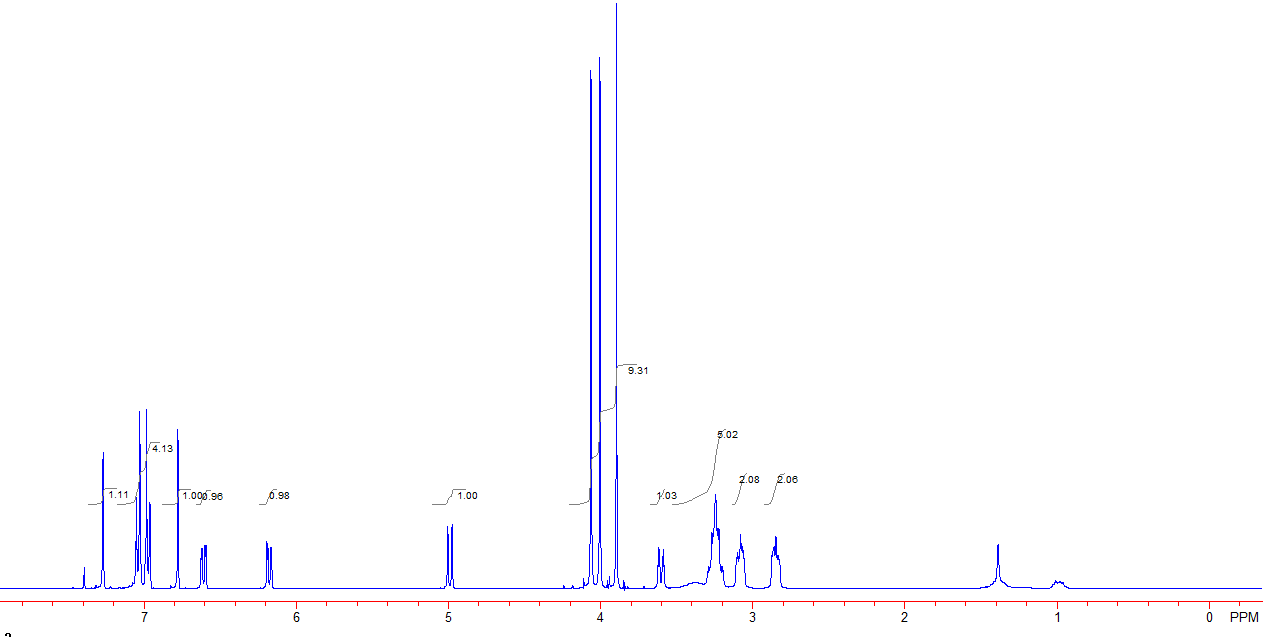


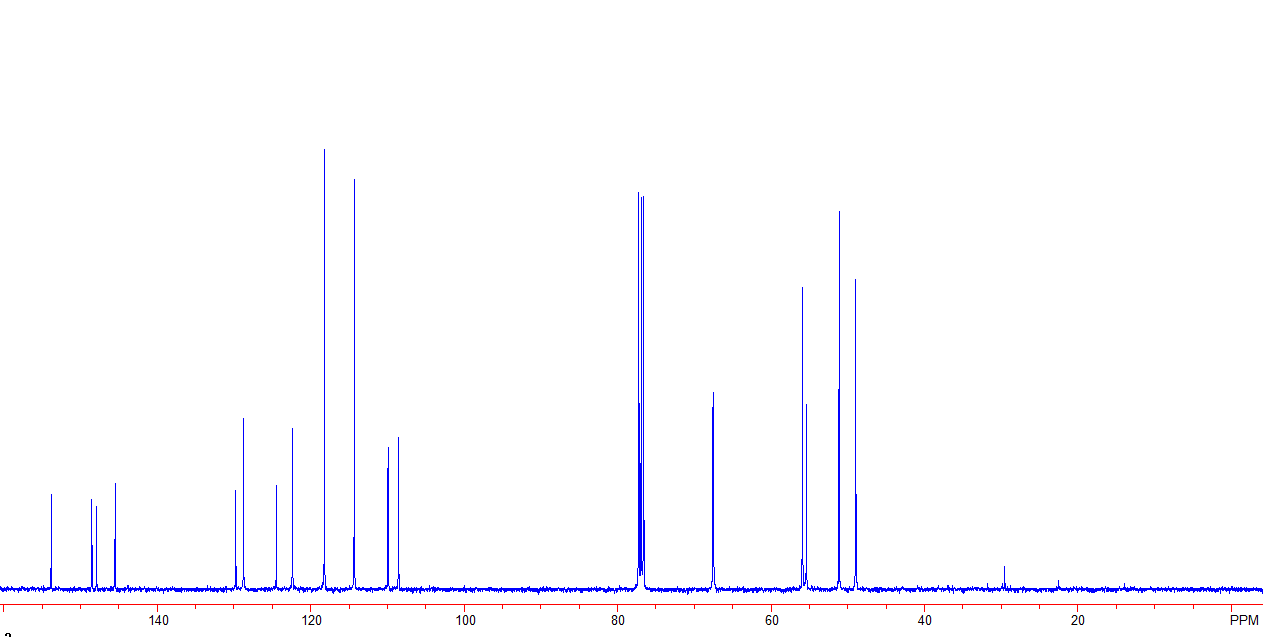


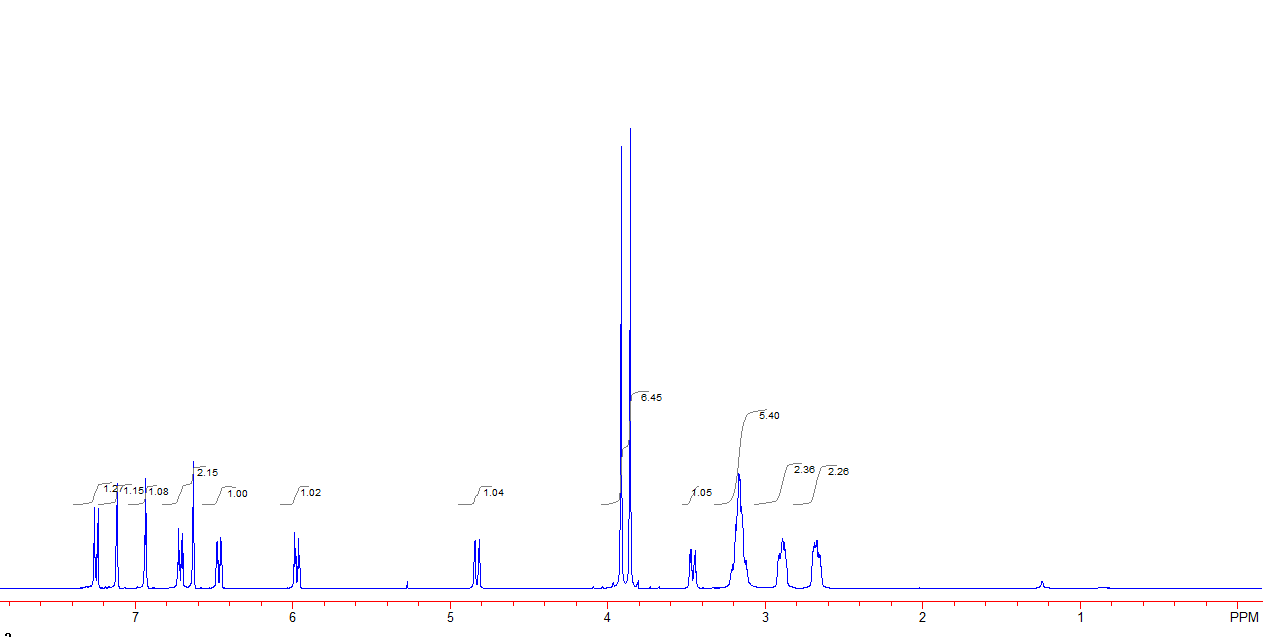


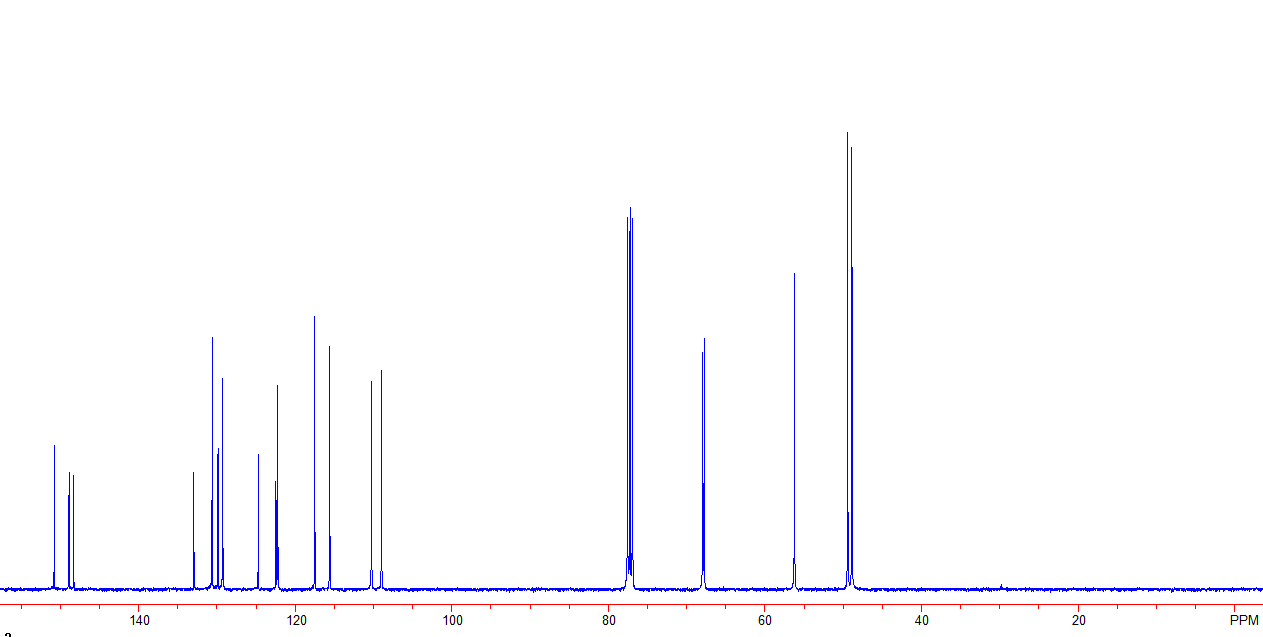

Supplement: File 1 — Experimental and analytical data [file Beilstein_J_Org_Chem-05-53-s001.doc]
